# Neonatal and child mortality data in retrospective population-based surveys compared with prospective demographic surveillance: EN-INDEPTH study

**DOI:** 10.1186/s12963-020-00232-1

**Published:** 2021-02-08

**Authors:** Tryphena Nareeba, Francis Dzabeng, Nurul Alam, Gashaw A. Biks, Sanne M. Thysen, Joseph Akuze, Hannah Blencowe, Stephane Helleringer, Joy E. Lawn, Kaiser Mahmud, Temesgen Azemeraw Yitayew, Ane B. Fisker, and the Every Newborn-INDEPTH Study Collaborative Group, Peter Byass, Joy E. Lawn, Peter Waiswa, Hannah Blencowe, Judith Yargawa, Joseph Akuze, Ane B. Fisker, Justiniano S. D. Martins, Amabelia Rodrigues, Sanne M. Thysen, Gashaw Andargie Biks, Solomon Mokonnen Abebe, Tadesse Awoke Ayele, Telake Azale Bisetegn, Tadess Guadu Delele, Kassahun Alemu Gelaye, Bisrat Misganaw Geremew, Lemma Derseh Gezie, Tesfahun Melese, Mezgebu Yitayal Mengistu, Adane Kebede Tesega, Temesgen Azmeraw Yitayew, Simon Kasasa, Edward Galigawango, Collins Gyezaho, Judith Kaija, Dan Kajungu, Tryphena Nareeba, Davis Natukwatsa, Valerie Tusubira, Yeetey A. K. Enuameh, Kwaku P. Asante, Francis Dzabeng, Seeba Amenga Etego, Alexander A. Manu, Grace Manu, Obed Ernest Nettey, Sam K. Newton, Seth Owusu-Agyei, Charlotte Tawiah, Charles Zandoh, Nurul Alam, Nafisa Delwar, M. Moinuddin Haider, Md. Ali Imam, Kaiser Mahmud, Angela Baschieri, Simon Cousens, Vladimir S. Gordeev, Victoria Ponce Hardy, Doris Kwesiga, Kazuyo Machiyama

**Affiliations:** 1 IgangaMayuge Health and Demographic Surveillance System, Makerere University Centre for Health and Population Research, Iganga, Uganda; 2 Kintampo Health Research Centre, Kintampo, Ghana; 3 Health Systems and Population Studies Division, icddr,b, Dhaka, Bangladesh; 4 Dabat Research Centre Health and Demographic Surveillance System, Dabat, Ethiopia; 5 Department of Health Services Management and Health Economics, Institute of Public Health College of Medicine and Health Sciences, University of Gondar, Gondar, Ethiopia; 6 Bandim Health Project, Bissau, Guinea-Bissau; 7 Research Centre for Vitamins and Vaccines, Statens Serum Institut, Copenhagen, Denmark; 8 Bandim Health Project, OPEN, Institute of Clinical Research, University of Southern Denmark, Odense, Denmark; 9 Maternal, Adolescent, Reproductive & Child Health (MARCH) Centre, London School of Hygiene & Tropical Medicine, London, UK; 10 Department of Health Policy, Planning and Management, Makerere University School of Public Health, Kampala, Uganda; 11 Centre of Excellence for Maternal Newborn and Child Health Research, Makerere University, Kampala, Uganda; 12 Division of Social Science, New York University-Abu Dhabi, Abu Dhabi, United Arab Emirates

**Keywords:** Child mortality, Infant mortality, Neonatal mortality, Survey

## Abstract

**Background:**

Global mortality estimates remain heavily dependent on household surveys in low- and middle-income countries, where most under-five deaths occur. Few studies have assessed the accuracy of mortality data or determinants of capturing births in surveys.

**Methods:**

The Every Newborn-INDEPTH study (EN-INDEPTH) included a large, multi-country survey of women aged 15–49 interviewed about livebirths and their survival status in five Health and Demographic Surveillance Systems (HDSSs). The HDSSs undertake regular household visits to register births and deaths for a given population. We analysed EN-INDEPTH survey data to assess background factors associated with not recalling a complete date-of-birth. We calculated Kaplan-Meier survival estimates for both survey and HDSS data and describe age-at-death distributions during the past 5 years for children born to the same women. We assessed the proportion of HDSS-births that could be matched on month-of-birth to survey-births and used regression models to identify factors associated with matching.

**Results:**

69,176 women interviewed in the survey reported 109,817 births and 3064 deaths in children under 5 years in the 5 years prior to the survey. In the HDSS data, the same women had 83,768 registered births and 2335 under-five deaths in the same period. A complete date-of-birth was not reported for 1–7% of survey-births. Birthdates were less likely to be complete for dead children and children born to women of higher parity or with little/no education. Distributions of reported age-at-death indicated heaping at full weeks (neonatal period) and at 12 months. Heaping was more pronounced in the survey data. Survey estimates of under-five mortality rates were similar to HDSS estimates of under-five mortality in two of five sites, higher in the survey in two sites (15%, 41%) and lower (24%) in one site. The proportion of HDSS-births matched to survey-births ranged from 51 to 89% across HDSSs and births of children who had died were less likely to be matched.

**Conclusions:**

Mortality estimates in the survey and HDSS were not markedly different for most sites. However, neither source is a “gold standard” and both sources miss some events. Research is required to improve capture and accuracy to better track newborn and child survival targets.

## Key findings

**Table:** 

**What is new?**	
• **What was known already**: To fill data gaps, child mortality in low- and middle-income countries is mainly estimated based on retrospective survey data. Opportunities to compare these estimates to other data sources have been limited. • **What was done:** 69,176 women in five Health and Demographic Surveillance System (HDSS) sites were interviewed, reporting 109,817 births and 3064 deaths of children who were under 5 years in the 5 years prior to the survey. For the same women, 83,768 births and 2335 under-5 deaths were recorded in the HDSS data. We assessed indicators of quality of child mortality data from the EN-INDEPTH survey and compared obtained mortality estimates to HDSS data. Through the linkage between the HDSS-births and the EN-INDEPTH survey data, we identified child and woman level background factors associated with identifying an HDSS birth in the survey data.	
**What was found?**	
• **Data quality (survey vs HDSS):** o HDSS-recorded births of children who had died were less likely to be identified in the survey data than births of children who were alive. Similarly, children born > 2 years ago, children born to women with little or no education, born to women of higher parity and poorer women were less likely to be identified in the EN-INDEPTH survey. The sex of the child was not associated with identification in any of the sites. o The estimated levels of mortality differed by the source of information and site; the HDSS estimate was lower than the EN-INDEPTH survey in Kintampo and Dabat, higher in Bandim and similar in the other two sites. In all sites, the HDSS data contained fewer recorded births and deaths than the EN-INDEPTH data, but the relative difference was larger for deaths than for births in the four sites, which assume full information on all births. o Our data indicate that births and to a larger extend deaths are likely to be missed in retrospectively collected data, both when information is obtained for the past 5 years as in the EN-INDEPTH survey, but also through the HDSS data when recall periods become long. • **Data quality (recall and heaping):** o In the EN-INDEPTH survey, women recalled a complete date-of-birth for 93–99% of children born within the past 5 years. The recalled birthdate was less likely to be complete if the child had died. o In the EN-INDEPTH survey, there was marked heaping of age-at-death by weeks during the neonatal period and by 12 months in the four African sites, but not in Matlab HDSS, Bangladesh.	
**What next in measurement and research?**	
• **Measurement now:** These data gaps may lead to under-estimation of child mortality and data sources require improvement. • **Research needed:** Further investigation of accuracy, omissions and associated factors may improve estimation methods in both survey and HDSS data.	

## Background

Survey data obtained through Demographic and Health Surveys (DHS) [1] or Multiple Indicator Cluster Surveys (MICS) [2] are the main sources of information on child mortality in low- and middle-income countries, which carry most of the burden of the estimated 5.3 million annual child deaths [3]. In DHS/MICSs, women aged 15–49 are interviewed about the children they have given birth to and the survival status of each child. If the child has died, the age-at-death is registered. For each child, women are asked to state the date-of-birth, which allows placing events in calendar time. These data are key to child mortality estimates, since almost two thirds of the global deaths are not captured through the civil registration and vital statistics [4].

Since the survey data fill gaps where civil registration and vital statistics systems do not generate accurate measures of mortality levels, opportunities to compare estimates have been limited. However, two studies have investigated how the retrospective survey estimates correspond to longitudinally collected mortality information obtained through regular home visits in Health and Demographic Surveillance System (HDSS) sites. In Bangladesh in 1994, mortality data were collected through a DHS-type survey targeting a sample of women also followed through the Matlab HDSS, Bangladesh. The authors report that while the completeness of live births (proportion of livebirths known through the HDSS also captured in the survey) was 99%, the completeness of infant deaths captured in the survey was only 84%, with early neonatal deaths (days 0–6) being particularly likely to be missed (completeness 80%) [5]. In the IgangaMayuge HDSS in Uganda, the HDSS estimates of child mortality were also higher than the estimates obtained through the survey methodology in the same area [6].

DHS/MICS data are used to produce estimates of early neonatal, neonatal, infant and under-5 mortality; the estimates are based on the number of children dying before 7 days, 28 days, 1 year and 5 years respectively among children under risk in the relevant age and calendar time window. Age heaping, the phenomenon that the reported age-at-death is shifted from the actual age to a particular age is known to occur at full weeks [7, 8], months and around 12 months of age [9]. It is important for the estimate of early neonatal mortality, since misclassifying a day 5–6 death as having occurred at 1 week shifts the death out of the early neonatal window. Similarly, classifying deaths occurring during the 4th week of life as having occurred at 1 month, or late infant deaths as having occurred at 1 year, shifts deaths out of the neonatal and infant windows. Inaccuracy in reported age-at-death around 5 years of age has less impact on estimated under-5 mortality, since mortality for 4–5-year-old children, and thus, the number of deaths, which may be shifted, is lower.

To lessen the impact of age heaping, global estimates of infant and neonatal mortality are based on models where the measured levels of under-5 mortality are an input. Previously, the models have been based on assumed proportions of under-5 deaths within infancy [10]. However, as also noted by others [11], predicted rates may not appropriately describe mortality patterns. Currently, estimates are based on modelled ratios of neonatal to under-5 mortality as well as country-specific input data [12]. These models allow greater flexibility but also emphasise the need for empirical data.

In 2017–2018, we conducted the Every Newborn-International Network for the Demographic Evaluation of Populations and their Health (EN-INDEPTH) study including a cross-sectional, multi-site randomised comparison in five HDSS sites within the INDEPTH network [13]. The primary objective of the EN-INDEPTH study was to randomly compare two methods of retrospective recording of pregnancy outcomes: a full birth history with additional questions on pregnancy losses (FBH+), as per the current standard in DHS-7, and a full pregnancy history (FPH). Details of the study protocol and the results of the primary objective have been published elsewhere [14, 15].

Nesting this study in HDSS sites with prospective follow-up of pregnancies and births provided an opportunity to compare the mortality patterns assessed through the longitudinal HDSS surveillance and the retrospectively captured mortality data from the EN-INDEPTH survey modules.

This paper is part of a series of papers from the EN-INDEPTH study. The aim of this paper is to use the data on livebirths collected as part of the EN-INDEPTH study and in the corresponding HDSS data to describe the quality of child mortality data in population-based surveys. The objective of the paper is to assess indicators of data quality for childbirth/death data collected through a survey, by describing:

1. ***Reported precision***: describing the proportion of women reporting a known date-of-birth (FBH+, FPH) and where applicable a date of death (FPH) and identify factors associated with not reporting a complete date-of-birth/death in the EN-INDEPTH survey.
2. ***Consistency of mortality estimates***: identify discrepancies in levels and age distributions of child mortality, including an assessment of heaping, between the EN-INDEPTH survey and the HDSS data.
3. ***Capture of births***: measure the proportion of HDSS-recorded births identified in the EN-INDEPTH survey and describe background factors associated with identifying an HDSS-registered birth in the survey.

## Methods

### Overall study design and setting

The EN-INDEPTH study included the following HDSS sites: Bandim in Guinea-Bissau, Dabat in Ethiopia, IgangaMayuge in Uganda, Matlab in Bangladesh and Kintampo in Ghana. A population-based survey of women of reproductive age was undertaken between July 2017 and August 2018. The EN-INDEPTH survey included 69,176 women across the five sites [15]. The survey questions were based on DHS-7 and were administered using tablets with data collected in Survey Solutions [16]. A sample of women aged 15–49 years (Dabat and IgangaMayuge) or a sample of women aged 15–49 with a registered birth within the last 5 years (Bandim, Matlab and Kintampo) were interviewed (Additional file 1.1) [14]. Dependent on which questionnaire the woman was randomised to be interviewed with, women provided information on all live births in their lifetime and pregnancy losses in the past 5 years (FBH+: 34,805 women), or information on all pregnancies in their lifetime regardless of the birth-outcome (FPH: 34,371 women). Randomisation to one of the two different questionnaires was performed using a build-in function evoked after the consent process [15]. The survey data collection is described in details elsewhere [14, 15], and only data on reported live births are used for the present analyses. In addition to the pregnancy or birth history, women were interviewed about their educational status and completed a detailed interview on house-construction materials and possessions, which allowed the generation of a wealth index.

All five HDSS sites conduct regular home visits to the population under study to register pregnancies and to follow-up on registered pregnancies and children. While the principles of surveillance are the same across all sites, the visit frequency, size and methods for capturing past events differ between sites (Additional file 1.1, 1.2 and 1.3).

### Methods by objective

#### Objective 1: reported precision: describe the completeness of dates of childbirth and death and factors associated with not reporting complete birthdates

In both the FBH+ and the FPH interview, women were requested to state the date-of-birth of all their live-born children. In addition, for children who had died, age-at-death was recorded in days if <1 month, in months if <2 years and in years if ≥2 years. In addition, women interviewed using the FPH were asked to state the date of death of the child. For recorded birth and death dates, an incomplete date (i.e. only year and month or only year) could be recorded by the interviewer if the mother could not state a complete date. We assess the proportion reporting an incomplete date. We explored the background factors associated with incomplete birthdates in binomial regression models with robust standard errors allowing for intragroup correlations within the same mother. We tested whether the association differed by site by including an interaction term in the model.

#### Objective 2: consistency of mortality estimates: identify discrepancies in levels and age distributions of child mortality, including an assessment of heaping, between the EN-INDEPTH survey and the HDSS data

In each site, for all live-born children born to the women interviewed in the EN-INDEPTH survey, we estimated early neonatal, neonatal, infant and under-5 mortality in the past 5 years using Kaplan-Meier survival estimates in both sources of data overall, and for women who in the EN-INDEPTH survey stated that they had continuously lived in the present city/town/village for the past 5 years (Additional file 1.3). In the EN-INDEPTH survey data, all children were considered at risk from the date-of-birth. In the HDSS data, we used delayed entry to allow for left-censored data, as not all children have been under HDSS surveillance since birth and death before registration would not have been captured in the HDSS. Since the data collection methodology differs by HDSS site, we used the HDSS definitions of when children were under surveillance. In four of the sites, children born in the area/born to women registered in the HDSS were considered part of the population since date-of-birth, while in Bandim HDSS, children were part of the population from birth only if the pregnancy was registered (Additional file 1.1 and 1.2). Observation time was censored at the date of EN-INDEPTH survey interview, except for Dabat HDSS, where we censored on September 4, 2017, as later data were not available for analysis (Additional file 1.3).

We graphically described the age distribution of mortality in both sources of data by plotting the proportion of mortality reported to have occurred in a particular interval (daily for the neonatal period and monthly for the first 2 years of life) by site and background factors (Additional file 1.3). Graphs for the neonatal period were produced in- and excluding day 0–1. Heaping at 7 days and 12 months was quantified by calculating the heaping index [8], describing how many times the number of deaths on day 7/month 12 was higher than expected with the expected number being the average of day 5–9 /month 10–14 (Additional file 1.3).

#### Objective 3: capture of births: identify factors (including survival status) associated with capturing an HDSS-recorded birth in the EN-INDEPTH survey data

While neither of the datasets may hold the perfect data on all births, most events in the HDSS data should be recorded with higher accuracy due to bi-annual or more frequent data collection rounds. Under the assumption that the date-of-birth obtained through the HDSS is correct, the proportion of births identified also in the survey data is a measure of completeness. We linked the dataset of registered HDSS-children contributing survival time during the past 5 years and born to interviewed women to the corresponding survey data. Linkage was performed on the mothers’ ID, and we assess the probability of an HDSS-birth being identified through the survey. We considered an HDSS birth matched to a survey birth if the mother during the survey reported a birth within 1 month of the HDSS-recorded birthdate. For births in the survey reported with only month and year, we relaxed the matching criteria to +/−2 months. For births in the survey where only year was reported, we further relaxed the matching criteria to only year of birth. In binomial regression models with robust standard errors allowing for intragroup correlations within the same mother, we identified factors associated with the capture of a birth in the survey data, including whether capture depended on whether the child subsequently died. In sensitivity analyses, we assessed whether (a) limiting the analysis to births registered already during pregnancy and (b) relaxing the criteria for a match to HDSS birthdate +/−3 and +/−9 months affected the conclusions.

All analyses were undertaken using Stata 16.0, and regression analyses excluded observations with missing information on the independent variables. Results are reported in accordance with STROBE Statement checklists for cross-sectional studies [17] (Additional file 2).

## Results

**Fig. 1:**
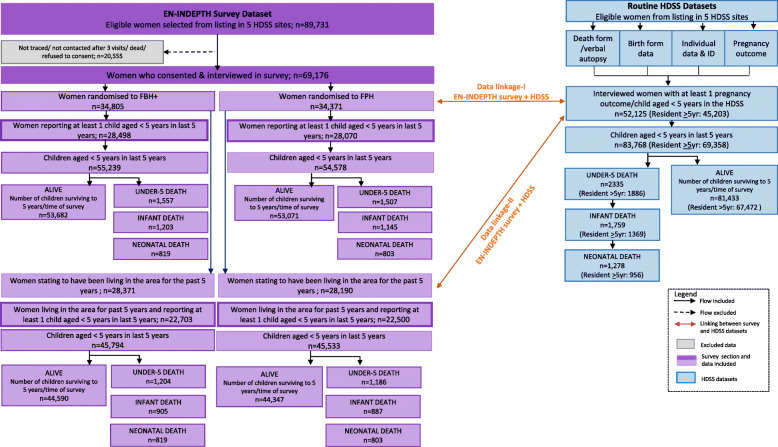
Flow diagram of the EN-INDEPTH study population showing data included for mortality analyses

Among the 69,176 women interviewed in the EN-INDEPTH study (Fig. 1), 59,638 reported having given birth to one or more live-born children (median number ranging from 2 in Matlab to 4 in IgangaMayuge and Dabat). A total of 195,021 live births were reported (Additional file 3.2). For births within the past 5 years, the proportion of children for whom the women did not recall a complete date-of-birth differed by site: 7% in Bandim and Kintampo, 6% in IgangaMayuge, 2% in Matlab and 1% in Dabat (Table 1). Birthdates were more likely to be incomplete for births that occurred more than 2 years prior to the survey, for children who had died and for children born to women with higher parity, while women with higher education and in higher wealth quintiles (with the exception of Bandim), were less likely to report incomplete birthdates. Sex of the child was not associated with whether a date-of-birth was recalled (Table 1). Complete date-of-birth for children born more than 5 years prior to the survey and dates of death for children born in the last 5 years were recalled for fewer children (Additional file 3.2).

**Table 1:** Factors associated with not recalling a complete date-of-birth among children <5 years contributing survival time

	Bandim	Dabat	IgangaMayuge	Matlab	Kintampo	RR (95%CI) adjusted for HDSS site	***P*** value for interaction with site^**a**^
Number of births	18423	16029	16044	32308	27013		
Incomplete date-of-birth^b^	7% (1376/18423)	1% (146/16029)	6% (1010/16044)	2% (755/32308)	7% (1832/27013)		
**Recall period**							
Born within past 2 years	2% (116/5045)	0% (12/3366)	2% (73/3426)	1% (57/8322)	2% (99/5572)	1 (ref)	0.39
Born more than 2 years ago	9% (1260/13378)	1% (134/12663)	7% (937/12618)	3% (698/23986)	8% (1733/21441)	4.08 (3.67–4.55)	
**Survival status**							
Alive	6% (1129/17657)	1% (133/15639)	6% (898/15567)	2% (650/31591)	6% (1523/26299)	1 (ref)	< 0.001
Died before 5 years	32% (247/766)	3% (13/390)	23% (112/477)	15% (105/717)	43% (309/714)	5.82 (5.42–6.24)	
**Sex**							
Male	7% (682/9413)	1% (71/8026)	6% (511/8075)	2% (368/16044)	7% (914/13736)	1 (ref)	0.89
Female	7% (631/8947)	1% (75/8003)	6% (499/7969)	2% (386/16263)	7% (918/13277)	1.01 (0.96–1.07)	
**Education**							
One	15% (996/6506)	1% (128/11113)	15% (246/1621)	9% (124/1407)	9% (1077/11918)	1 (ref)	< 0.001^c^
Primary school	5% (286/5475)	1% (17/3083)	7% (589/9049)	5% (344/6338)	5% (745/14142)	0.47 (0.44–0.51)	
Secondary school	2% (84/5364)	0% (1/859)	3% (162/4703)	1% (285/20679)	1% (9/701)	0.15 (0.13–0.17)	
Higher education	1% (8/1074)	0% (0/974)	2% (13/671)	0% (2/3884)	0% (1/252)	0.04 (0.02–0.06)	
Missing	50% (2/4)						
**Parity**							
1	2% (48/2426)	0% (2/1259)	2% (27/1150)	1% (33/6345)	2% (40/1944)	1 (ref)	< 0.001
2	5% (193/3765)	0% (8/2218)	3% (52/1874)	1% (187/13040)	5% (202/3917)	2.33 (1.95–2.79)	
3	7% (275/3817)	1% (15/2310)	3% (61/2171)	3% (245/8511)	6% (306/4847)	3.52 (2.95–4.20)	
4	9% (263/3048)	1% (13/2505)	4% (90/2323)	6% (179/3147)	7% (326/4807)	4.41 (3.67–5.29)	
5+	11% (597/5367)	1% (108/7737)	9% (780/8526)	9% (111/1265)	8% (958/11498)	6.00 (5.05–7.14)	
**Wealth quintiles**							
Poorest	4% (127/3344)	1% (30/4644)	8% (336/4461)	4% (307/6877)	8% (482/5956)	1 (ref)	< 0.001
2	6% (211/3430)	1% (39/3281)	7% (252/3618)	3% (205/6476)	8% (467/5635)	1.00 (0.90–1.10)	
3	8% (285/3537)	1% (48/3450)	7% (223/3012)	2% (119/6355)	7% (354/5402)	0.92 (0.83–1.02)	
4	8% (274/3608)	1% (26/2679)	5% (137/2748)	1% (76/6274)	6% (312/5167)	0.76 (0.68–0.85)	
Richest	11% (479/4504)	0% (3/1975)	3% (62/2205)	1% (48/6326)	4% (217/4853)	0.75 (0.67–0.83)	

^a^Interaction with HDSS site tested in a separate model

^b^Complete date-of-birth defined as day, month and year of birth provided. An incomplete date-of-birth includes all dates of birth missing at least one of day, month and/or year

^c^Tested by grouping secondary and higher education together

### Mortality data by source of information

A total of 109,817 children contributing observation time before 5 years of age within the past 5 years were registered to 56,568 women (Fig. 1, Table 2). Among these children, 3064 (2.8%) died before 5 years of age. The survey-estimated under-5 mortality rate among the women interviewed ranged from 35 per 1000 live births in Matlab to 67 per 1000 live births in Bandim (Table 3). Among the women with children contributing observation time, 27–32% reported having children who did not live with them in the African sites, while this was only 9% in Matlab. Limiting the analysis to women who in the survey did not report any children not contributing survival time in the past 5 years, the proportions were 2–12% (Additional file 3.4).

**Table 2:** Summary of EN-INDEPTH and HDSS data for children born to interviewed women

	Bandim (Guinea-Bissau)		Dabat (Ethiopia)		IgangaMayuge (Uganda)		Matlab (Bangladesh)		Kintampo (Ghana)	
	Survey	HDSS	Survey	HDSS	Survey	HDSS	Survey	HDSS	Survey	HDSS
**Number of women interviewed**	9492		12,593		13,437		21,462		12,192	
Number of women with children contributing survival information (% of interviewed)^a^	9319 (99%)	9184 (97%)	7781 (87%)	4933 (55%)	6901 (84%)	5929 (86%)	20,484 (97%)	20,446 (95%)	12,083 (99%)	11,633 (96%)
Number of children contributing survival time^a^	18,423	16,191	16,029	7999	16,044	12,401	32,308	31,992	27,013	15,185
Number of under-five deaths^a^	766	753	390	166	477	352	717	668	714	396
Number of infant deaths^a^	624	636	326	122	350	216	554	500	494	285
Number of neonatal deaths^a^	432	480	212	68	225	150	451	403	302	177
Ratio of HDSS/survey births	0.88		0.50		0.77		0.99		0.56	
Ratio of HDSS/survey deaths	0.98		0.43		0.74		0.93		0.55	
Ratio of ratios^b^	1.12		0.85		0.95		0.94		0.99	
**Number of women interviewed stating to have lived in area** **>** **5 years**	5895 (62%)		11476 (91%)		11854 (88%)		16252 (76%)		11084 (91%)	
Number of women with children contributing survival information (% of interviewed)^a^	5773 (98%)	5706 (97%)	7160 (62%)	4671 (41%)	5714 (48%)	5011 (42%)	15570 (96%)	15556 (96%)	10986 (99%)	10566 (95%)
Number of children contributing survival time^a^	12,013	11,103	15,043	7,665	13,588	10,866	26,003	25,836	24,680	13,888
Number of under-five deaths^a^	489	526	351	158	382	322	525	509	643	371
Number of infant deaths^a^	403	433	289	114	266	194	391	363	443	265
Number of neonatal deaths^a^	276	317	184	61	167	134	308	282	271	162
Ratio of HDSS/survey births	0.92		0.51		0.80		0.99		0.56	
Ratio of HDSS/survey deaths	1.08		0.45		0.84		0.97		0.58	
Ratio of ratios^b^	1.16		0.88		1.05		0.98		1.03	

^a^During the past 5 years. Note that the sampling frame differed by HDSS site, among all women with a registered birth outcome in the past 5 years in Bandim, Matlab and Kintampo, but among all women in Dabat and IgangaMayuge

^b^Ratio of HDSS deaths/survey deaths divided by ratio of HDSS births/survey births

**Table 3:** Mortality estimates based on HDSS and EN-INDEPTH study

	Mortality estimates per 1000 live births among children born to surveyed women	
	HDSS estimate	EN-INDEPTH estimate
**Bandim**		
Early neonatal	51 (4656)	34 (31–37)
Neonatal	55 (51–60)	36 (33–40)
0–90 days	61 (56–66)	41 (37–44)
Infant	72 (67–77)	53 (49–58)
Under-3	82 (77–88)	65 (61–70)
Under-5^a^	88 (82–95)	67 (62–72)
**Dabat**		
Early neonatal	10 (7–13)	20 (17–24)
Neonatal	17 (13–22)	26 (23–30)
0–90 days	23 (19–28)	33 (29–37)
Infant	30 (25–35)	40 (36–44)
Under-3	39 (34–46)	47 (42–51)
Under-5^a^	41 (35–47)	47 (43–52)
**Iganga-Mayuge**		
Early neonatal	22 (19–26)	25 (22–29)
Neonatal	25 (22–30)	27 (24–30)
0–90 days	27 (23–31)	29 (26–33)
Infant	36 (32–41)	42 (38–46)
Under-3	52 (47–58)	53 (48–58)
Under-5^a^	57 (52–63)	56 (51–61)
**Matlab**		
Early neonatal	17 (15–19)	18 (17–20)
Neonatal	20 (18–22)	22 (20–24)
0–90 days	22 (20–24)	24 (22–26)
Infant	25 (23–27)	27 (25–29)
Under-3	32 (29–34)	34 (31–36)
Under-5	35 (32–38)	35 (33–38)
**Kintampo**		
Early neonatal	12 (10–14)	17 (15–19)
Neonatal	14 (12–17)	19 (17–22)
0–90 days	17 (15–20)	22 (20–25)
Infant	22 (20–25)	32 (29–34)
Under-3	31 (28–34)	44 (41–47)
Under-5	32 (29–36)	45 (42–49)

Early neonatal < 7 days, neonatal < 28 days and infant < 1 year. ^a^Under-3 mortality added as Bandim HDSS follow children below the age of 3 more intensively, and some deaths between 3 and 5 years may not yet have been registered through the HDSS

Among the 69,176 women interviewed in the EN-INDEPTH survey, 52,125 had one or more children who were registered and followed in the HDSS during the 5 years prior to the EN-INDEPTH survey. Among these 83,768 children, we had registered 2335 deaths. Using the HDSS definition of when a child is part of the population (Additional file 2.1), under-five mortality estimates ranged from 35 in Matlab to 88 in Bandim (Table 3).

While the number of children under surveillance during the 5 years prior to the survey was similar for the HDSS and EN-INDEPTH survey data in Matlab, the numbers in the HDSS data were markedly lower in the other HDSS sites (Table 2). The number of deaths was also lower, but to a larger extent in the four sites where children born to registered women are considered under surveillance from birth regardless of whether the pregnancy is registered or not. This resulted in a ratio between the number of deaths (HDSS/survey) and the number of births (HDSS/survey) below one. For Bandim, the ratio was 1.12, since the number of births in the HDSS was 12% lower than in the survey while the number of deaths was 2% lower (Table 2). Limiting the analysis to the women who during the survey stated that they had consistently been living in the same location for the past 5 years increased the ratios of HDSS/survey registered death to births (Table 2).

**Fig. 2:**
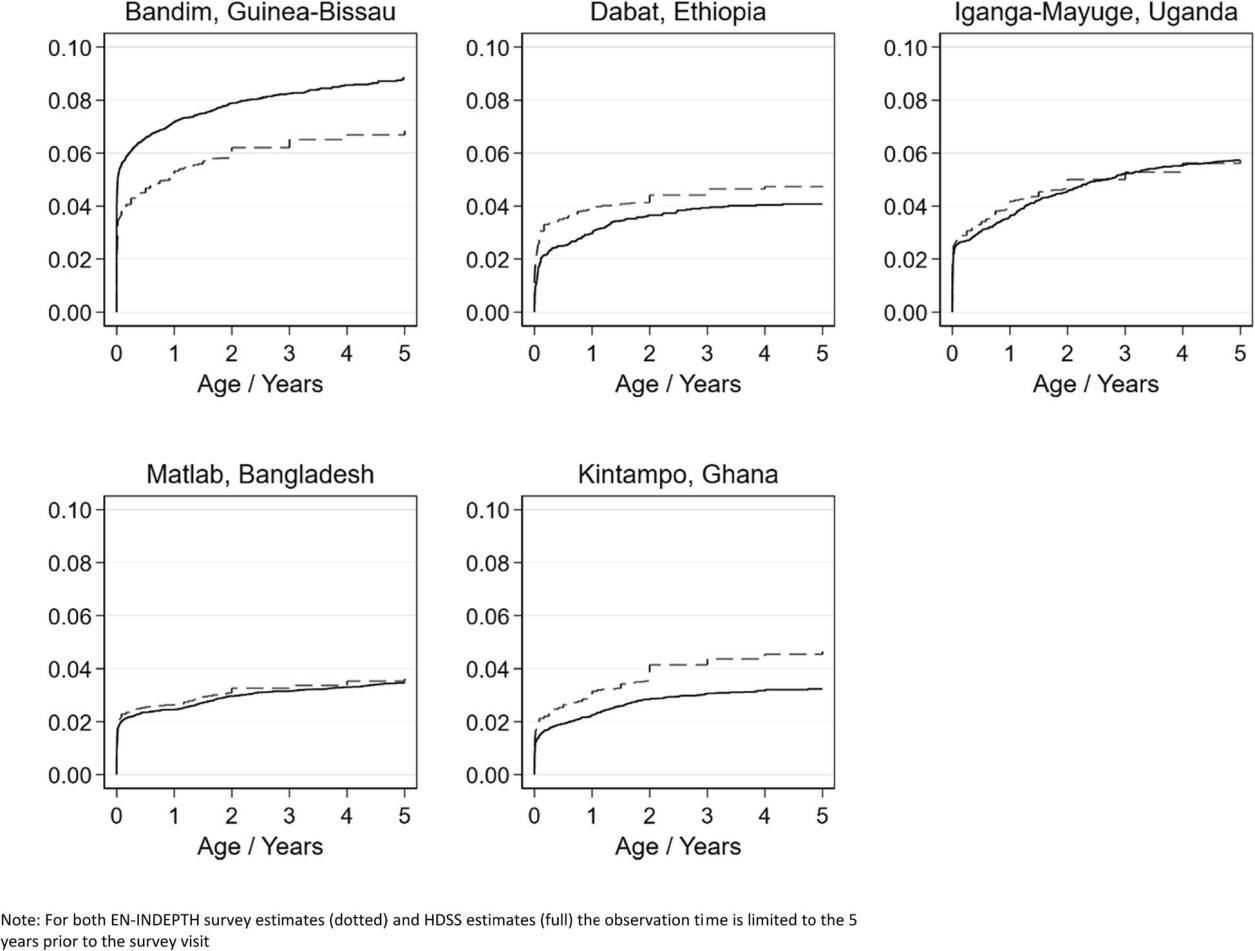
Cumulative mortality estimates among children born to women interviewed

Differences in the estimated mortality comparing the HDSS and the EN-INDEPTH survey data estimates were evident for Bandim, which had a 24% lower under-5 mortality in the survey compared with the HDSS and in Kintampo where the estimated under-5 mortality was 41% higher in the survey compared with the HDSS. Differences were much smaller for the other sites (Fig. 2, Table 3). Limiting the analysis to the women who had reported residence in the same location during the past 5 years revealed the same mortality pattern (Additional file 3.5). The proportion of under-5 mortality occurring in the neonatal period ranged from 43 to 54% in the survey data and from 42 to 63% in the HDSS data (Additional file 3.1).

### Age-at-death

**Fig. 3:**
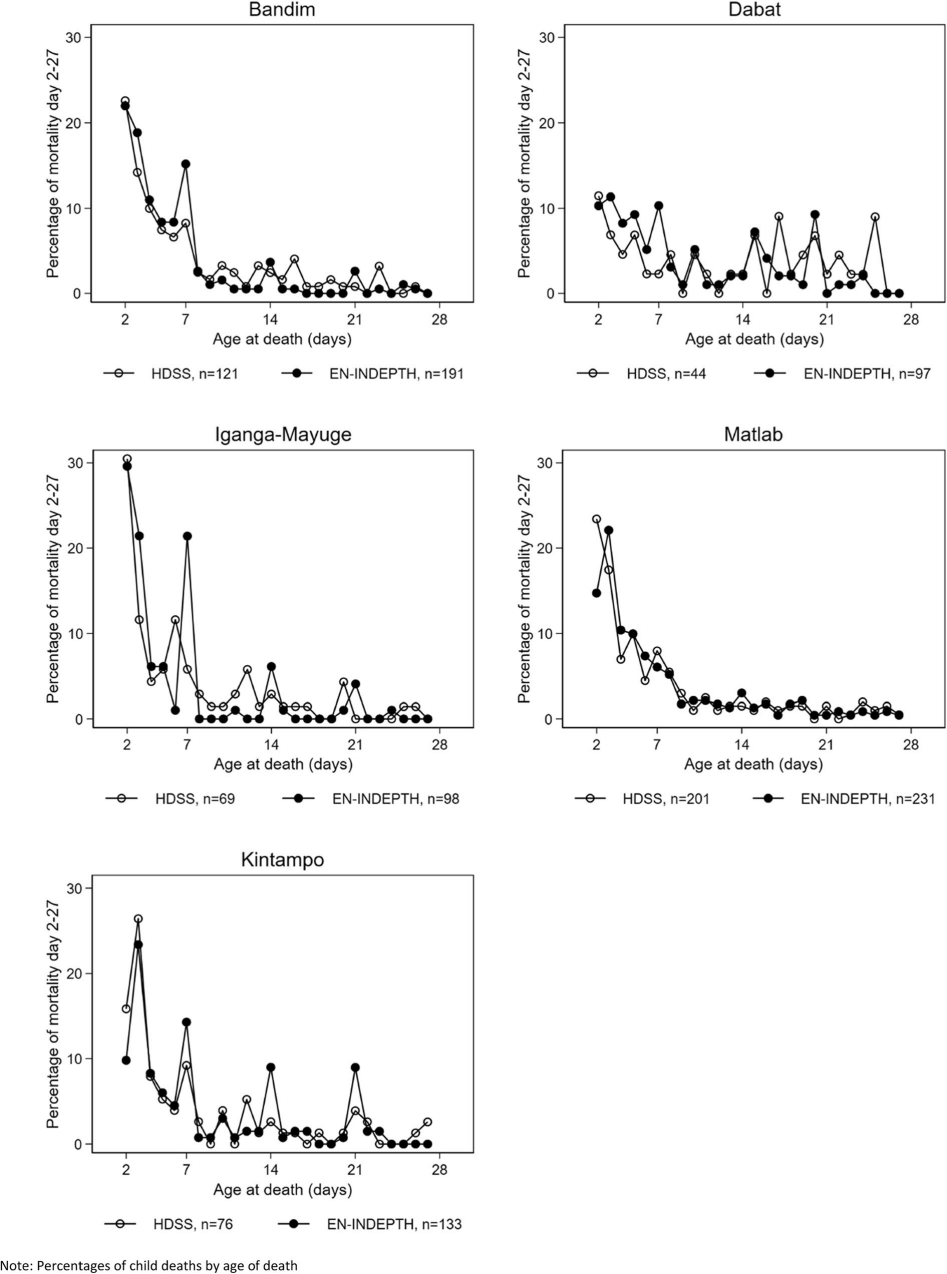
Distribution of mortality during the neonatal period (excluding day 0–1)

**Fig. 4:**
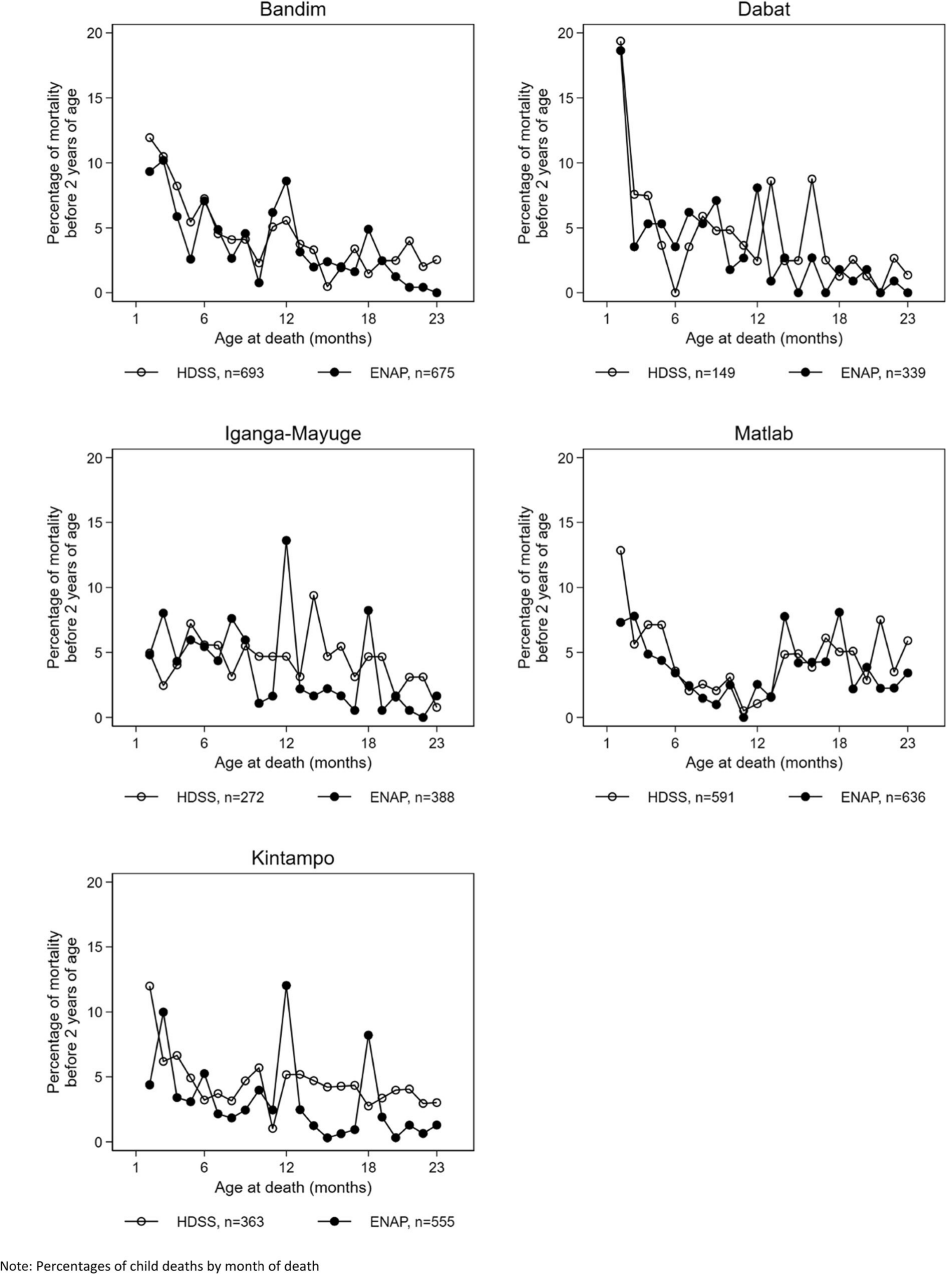
Distribution of mortality during the first 2 years of life

Age-at-death during the first month was reported in days, but a child who was born in the evening and died the subsequent morning, may have been classified as dead at day 1 rather than at age 0 days. Using Kaplan-Meier estimates, we have estimated mortality day 0–1 as mortality by day 1. Between 50 and 60% of the neonatal deaths were reported to have occurred day 0–1 in both survey and HDSS data (Additional file 3.6). After day 1, there was a preference for reporting age-at-death in full weeks in Bandim, IgangaMayuge and Kintampo, but not in Matlab. The Dabat data were more variable but based on small numbers (Fig. 3). Heaping indexes for death at 7 days of age in the African sites ranged from 1.8 (Dabat) to 3.8 (IgangaMayuge) for the survey data. In the HDSS data, the day-7-indices were lower, but there was still evidence of reporting preference (Table 4). In the survey data, heaping was also evident at 12 months in the African sites (range 2.1–3.4), but not in Matlab (heaping index 0.9). Heaping at 12 months was less pronounced in the HDSS data (Table 4, Fig. 4).

**Table 4:** Heaping index for deaths reported at 7 days and 12 months, EN-INDEPTH survey versus HDSS

	Bandim (Guinea-Bissau)		Dabat (Ethiopia)		IgangaMayuge (Uganda)		Matlab (Bangladesh)		Kintampo (Ghana)	
	Survey	HDSS	Survey	HDSS	Survey	HDSS	Survey	HDSS	Survey	HDSS
Deaths, day 7	29	10	10	1	21	4	14	16	19	7
Deaths, days 5–9	68	32	28	7	28	18	70	62	35	16
Heaping index day 7^a^	2.1	1.6	1.8	0.7	3.8	1.1	1.0	1.3	2.7	2.2
Deaths, 12 months	22	12	9	2	25	6	5	2	40	10
Deaths, 10–14 months	53	43	18	18	37	32	28	21	73	42
Heaping index 12 months^b^	2.1	1.4	2.5	0.6	3.4	0.9	0.9	0.5	2.7	1.2

^a^Heaping index estimated as number of death day 7/average number of deaths day between 5 and 9 days, i.e. deaths day 7/(deaths day 5–9/5)

^b^Heaping index estimated as number of death at 12 months/average number of deaths per month between 10 and 14 months, i.e. deaths 12 months/(deaths 10–14 months/5)

**Fig. 5:**
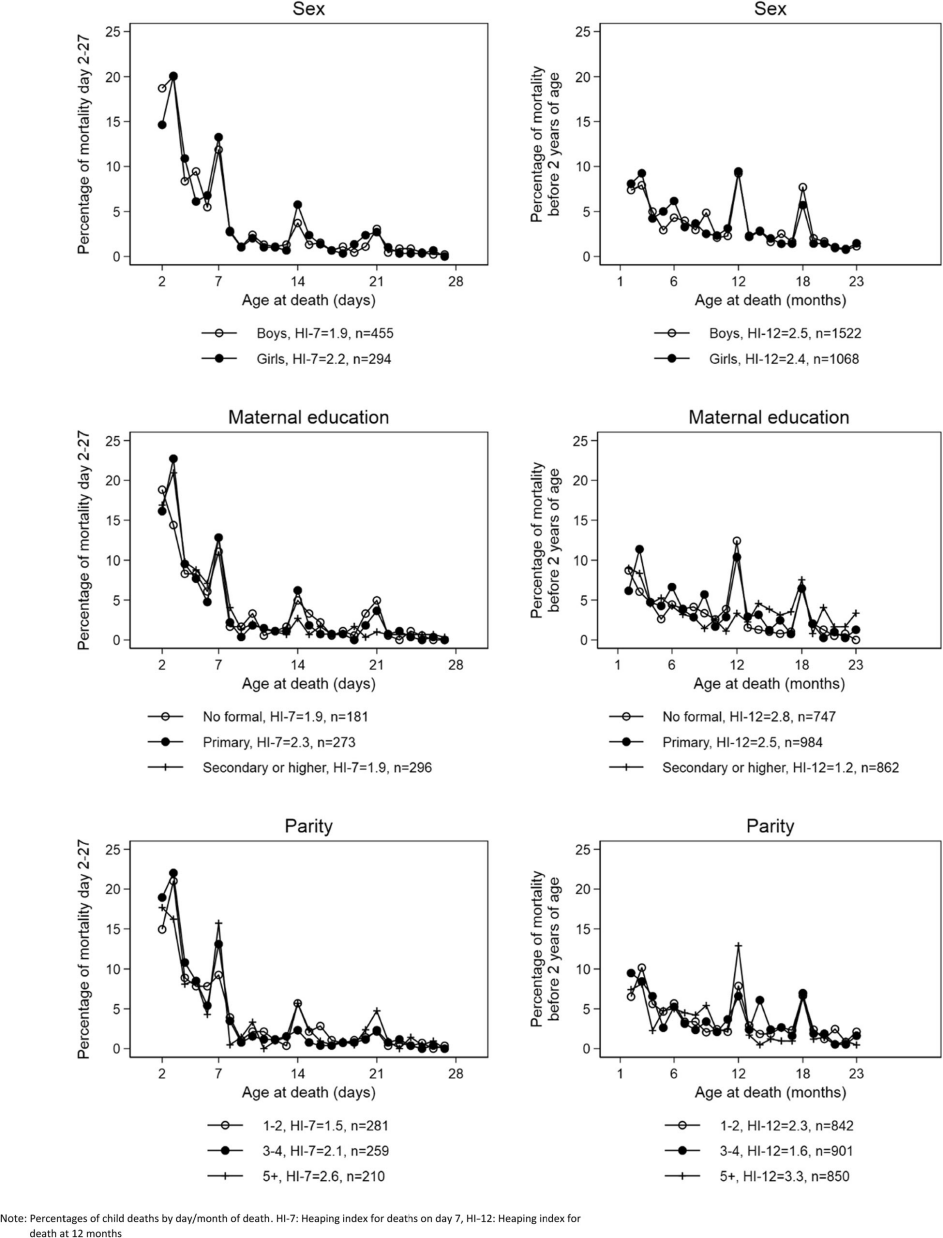
Distribution of mortality during the neonatal period (excluding day 0–1) and first 2 years of life

In the pooled EN-INDEPTH survey dataset, we found no marked differences in heaping by sex, parity (Fig. 5) and wealth quintile (Additional file 3.7). Our data indicate that heaping may be more pronounced for deaths among children of mothers with little or no education and mothers who had given birth to 5 or more children (Fig. 5).

### Factors associated with capturing an HDSS registered birth in the EN-INDEPTH survey data

**Fig. 6:**
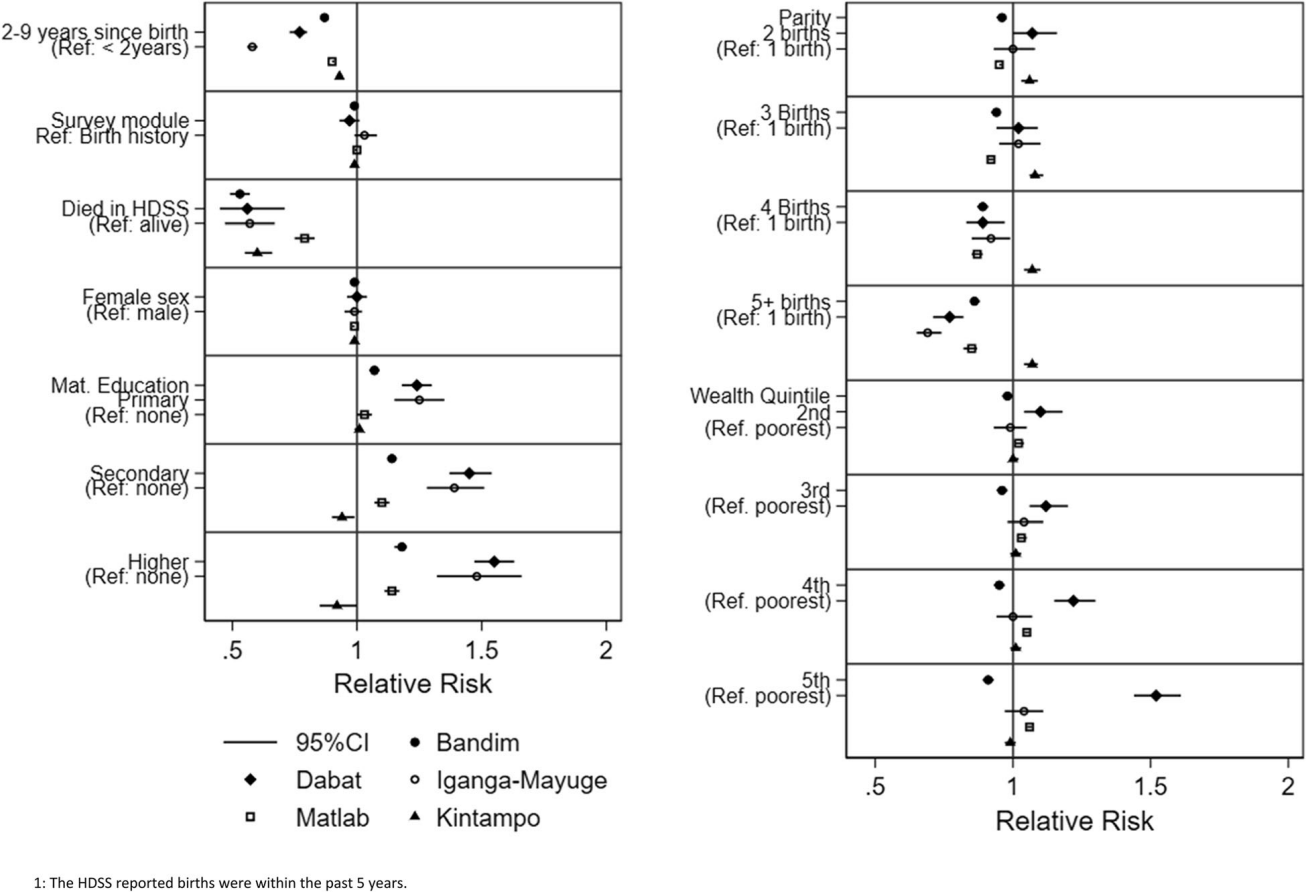
Relative risks of identifying an HDSS reported birth^1^ to an EN-INDEPTH survey recorded birth

When linking the HDSS-recorded births to the survey data, the proportion matched +/−1 month was 86% in Bandim, 58% in Dabat, 51% in IgangaMayuge, 89% in Matlab and 88% in Kintampo (Additional files 3.8A-3.8E). In all sites, a longer recall was associated with a lower probability of identification, but the magnitude differed widely by the site (Fig. 6, Additional files 3.8A-3.8E). Children who had died were less likely to be matched (Fig. 6); the estimates for the four African sites indicated a 40–47% lower probability of being matched while this estimate was 21% lower for Matlab (Additional files 3.8A-3.8E). Children born to more educated mothers and children of mothers who had given birth to fewer children were more likely to be matched in all sites with the exception of Kintampo (Additional files 3.8A-3.8E). The matching probability did not depend on sex or survey module in any of the sites. The wealth quintile was only associated with matching in Matlab and Dabat (Fig. 6). Limiting the attempted matched population to the children followed in the HDSS data since birth (73% of children in Bandim, 82% in IgangaMayuge, 91% in Dabat, 95% in Matlab and 99% in Kintampo) increased the proportion matched by 0–2 percentage points (Additional files 3.9A-3.9E), while relaxing the matching criteria to HDSS birthdate +/−3 and +/−9 months increased the proportion matched by up to 7 and 21 percentage points, respectively (Additional files 3.10A-3.10E and Additional files 3.11A-3.11E). The sensitivity analyses identified the same patterns of background factors associated with matching (Additional file 3.1, Additional files 3.9A-3.9E, Additional files 3.10A-3.10E and Additional files 3.11A-3.11E).

## Discussion

### Main findings

The proportion of children for whom a complete date-of-birth was recorded in the EN-INDEPTH survey data differed by site, but the regression analysis identified the same factors associated with recording an incomplete birthdate across all five sites. The date of death was less likely to be recalled than date-of-birth in all sites, but variation was large.

Despite comparable mortality rates in HDSS and survey data in three of the sites, the number of births and deaths differed markedly between the two data sources. Furthermore, the proportion of HDSS births matched to EN-INDEPTH survey data was considerably lower for children who had died than for children who had survived. Heaping of age-at-death at full weeks during the neonatal period and at 12 months was common in the EN-INDEPTH data, but less so in the HDSS data.

### Consistencies with other studies

Day of birth has only been reported in survey data since the introduction of DHS-7 in 2013 [18]. The proportion reporting imprecise birthdates has been described for two DHS-7 surveys: in Malawi (2015–2016), 4.6% birthdates were incomplete, while the proportion in Tanzania (2016–2017) was 1.6% [19]. Incomplete birthdates ranged from 1 to 7% in our sites, but the populations followed through HDSS sites may be more accustomed to reporting dates.

A prior study from IgangaMayuge indicates that the number of pregnancies identified in the year prior to the retrospective survey was higher than captured in the HDSS, although for longer recall periods, a higher number of pregnancies were missed through a retrospective pregnancy history survey [6]. A similar pattern for births may explain the linking pattern observed across sites here: births more than 2 years prior to the survey were less likely to be matched than births within the past 2 years. Lower matching rates for births of children who subsequently died than for children still alive have also been observed previously in Matlab [5]. In a survey collecting pregnancy and birth histories in Matlab in 1994, deaths which occurred >5 years ago and deaths at early ages were particularly likely to be omitted from the pregnancy survey [5]. Similar patterns for age-at-death were observed in the sites with the largest number of HDSS deaths (Bandim, Matlab and Kintampo) in the present study (Additional files 3.8A, 3.8D and 3.8E).

In addition to omission of births in the EN-INDEPTH survey, lack of matching could also be caused by displacement if births recorded in the EN-INDEPTH survey were reported to have occurred before or after the real date. While we have no direct measure of the displacement since the linking was at the level of the mother rather than the individual child, displacement has in a prior study been more common for children who had died [5].

In line with prior evidence, we observed that the age-at-death was heaped, preferentially reported at full weeks [7] and around 12 months of age [9] as reflected in peaks at 7, 14 and 21 days (Fig. 3) and at 12 months (Fig. 4). This heaping was only observed in the African sites and was more marked in the survey than HDSS data. While the lack of a 12-month peak in Matlab may be explained by the underlying different distribution of child mortality with child mortality increasing after 12 months of age due to drowning [20], this does not explain why there was less heaping in the neonatal period in this one site. The higher rate of maternal literacy and a lower number of children per woman in Matlab than in the other sites may explain why there is less heaping [21].

### Interpretation

The child mortality estimates from the five HDSS sites are not necessarily representative of the underlying child mortality in the HDSS, as the sample in three sites was not chosen at random to focus efforts on the women with births in the past 5 years [14]. Thus, the estimated mortality levels should not be interpreted as the HDSS mortality, but rather as the mortality levels for children born to the same subset of women.

The HDSS data do not include records of births that were never part of the HDSS population, i.e. HDSS data do not include births to interviewed women that occurred before the woman moved into the HDSS, where the child did not in-migrate with the mother since it had died or because the child was living elsewhere. In Dabat, the HDSS surveillance data were truncated approximately 18 months prior to the EN-INDEPTH survey. In the Bandim HDSS, only children followed prospectively in the HDSS data contribute time-at-risk in the mortality estimates, due to the assumption that deaths are less likely to be reported to the interviewers than surviving children [22] (Additional file 2.1, Additional file 2.2). Thus, the number of births and deaths in the HDSS data is a subsample of the real birth history of the women.

The number of HDSS births in this subset of women should thus be lower than the EN-INDEPTH data, which attempt to capture the full history of all live births, and the proportion registered should be lower in Bandim than the other sites. Looking at the Bandim numbers in Table 2, the ratio of HDSS birth to survey births of 0.92 among women resident in the same location for the past 5 years, does support that Bandim HDSS may capture only a sample of the births. However, as the other HDSSs seek to capture all births to resident women, even if the pregnancy had not been registered and the child would no longer be part of the population after registration, we expected that the ratios of HDSS to survey births would be higher in the other sites. With the exception of Matlab, this was not the case (Table 2). Censoring of the Dabat data likely explains much of the lower numbers in Dabat, but the 20 and 44% lower numbers in IgangaMayge and Kintampo in the HDSS compared with the survey among women stating residence in the same location for the past 5 years, are unlikely to be made up only by children who have never been living in the HDSS. Thus, some births are likely missed by the HDSS also in the sites, which assume full information of all births to resident women. If the proportion of HDSS-unrecorded children is independent of survival status, the number of deaths should be lower by a similar proportion as the number of births. In the four HDSSs, which assume full information of all births to women under HDSS surveillance, the ratio of the two ratios ‘deaths in HDSS to deaths in survey data’ vs ‘births in HDSS to birth in survey data’ was less than one (Table 2), which may indicate that under-reporting could be more severe for deaths. When limiting the analysis to women who had continuously lived at their present location for the past 5 years, this indication of relative underreporting of deaths to births in the HDSS relative to the survey was weakened in the four sites assuming full information on births to women under surveillance. In contrast, in Bandim, there was an indication that more deaths were captured in the HDSS than in the survey, the ratio of ratios being > 1.

A ratio of ratios of 1 either indicates that mortality is estimated correctly in both HDSS and survey data or that both estimates are off by a similar magnitude. Without a gold standard, making firm conclusions on either interpretation is not possible. However, when looking at the age distribution of under-5 mortality, the HDSS estimates indicate that 42, 44 and 44% of under-5 mortality was neonatal in Dabat, IgangaMayuge and Kintampo, respectively. The HDSS estimates were substantially higher in Matlab (57%), which had intensive surveillance with bimonthly visits and pregnancy testing after missed periods [23] and in Bandim (63%) (Additional file 3.1), where mortality estimates are based on prospective surveillance (Additional file 2.1). Thus some HDSSs also likely underestimate early mortality, especially when intervals between follow-up rounds are long: deaths in children under surveillance are captured, but early deaths among children born between rounds are likely to be missed [24].

Since the HDSS data are per definitions a subset of the real birth history, all HDSS-recorded births should have a matched birth record in the survey data had the precision of the birth dates been high in both sources. However, when the HDSS reported births were linked to the survey data (+/−1 months), only between 51% and 89% of the HDSS records were matched to child records in the EN-INDEPTH survey data. In all five sites, the probability of matching an HDSS birth to a birth in the EN-INDEPTH survey was lower if the child had died. This is consistent with the survey being more likely to miss births of children who have subsequently died and thus underestimates the real child mortality. However, as described above, misreported date-of-birth for children who died may also contribute.

### Strengths and limitations

This is to our knowledge the first study investigating variation in child mortality data measured through retrospective survey data across a range of countries and in populations where survey data could also be linked to prospective data on mortality at the level of births. In spite of having estimates from two different data sources, we do not have a gold standard. Some births and deaths may be missed through either or both sources, because they were not reported to the interviewer. Thus, there is no gold standard to evaluate either of the measures against.

Potentially, though the women to be interviewed were selected from a listing of women registered in the HDSS data, another woman may have been interviewed instead of the listed woman. Such errors may have occurred since the common way of identifying a woman in the HDSS sites is ‘mother of xx’; for the present listing, we could not use these relations and that may have hampered the identification. Nevertheless, this is likely to be rare and does not explain the difference in matching by survival status of the child.

### Implications

While the HDSS does not capture the true full birth history, our analyses indicate that the survey data likely missed some births too and in particular births where the child had subsequently died. Thus, both mortality from some HDSSs and estimates from surveys may systematically underestimate child mortality. Since the retrospective survey interviews are conducted to fill data gaps, studying omissions is challenging. We found that the EN-INDEPTH survey underestimated mortality compared with the Bandim HDSS, but we did not observe this pattern in the other sites, where full information of all births to registered women is assumed. In light of the different HDSS definitions of when a child is under surveillance (Additional file 2.1 and 2.2), future studies should ensure that both the data where full information on all births is assumed and the additional data necessary to perform analyses limited to prospective follow-up is available in the same populations. Sex-ratio at birth has been suggested as an indicator of potential omissions [25], but none of our analyses indicated that sex was associated with the likelihood of linking, which it should have been if girls or boys were selectively underreported. Sex-ratios alone are therefore not enough to reassure completeness of the survey data.

The precision of age-at-death is important in establishing the proportion of deaths having occurred below a specific age and thus to inform global mortality estimates [12]. With changes in mortality patterns, departures from previously modelled fractions of mortality in younger age groups may be introduced, but could be overlooked in the absence of empirical data. Thus, improved measurement of infant and neonatal mortality is necessary to monitor progress towards the mortality targets of the Sustainable Development Goals [26].

Establishing the precision of the survey data may, furthermore, open up for new use of this type of data. If the survey data is sufficiently accurate, DHS/MICS data could be useful in studying the effects of ‘shocks’—e.g. effects of environmental exposures, pandemics or other events fixed at specific time points both before and after birth, and therefore be relevant for targeting interventions. If the imprecision increases markedly with the recall period, assessing potential effects of events several years prior to the survey may be impossible. The consistent finding that the sex of the child was not associated with the indicators of precision opens up to the use of survey data to study interventions, which may affect boys and girls differently [27] or differences in access to care, which may cause sex-differential mortality patterns [28].

## Conclusions

Using survey estimates of child mortality in the absence of other data sources is a necessity, but our analyses indicate that estimates should be used with caution. Further investigation of accuracy, omissions and factors associated therewith may contribute to improve mortality estimates. While civil and vital registrations are being strengthened in many countries, surveys remain crucial for the countries with the highest mortality burden. More investment is needed to improve survey tools and their implementation.

## Supplementary information

**Additional file 1.** Additional methods. 1.1: Description of key differences between the HDSS sites. 1.2: Illustration of how observation time is counted in the EN-INDEPTH survey and different HDSS sites. 1.3: Additional methods for objective 2.

**Additional file 2.** STROBE guidelines checklist.

**Additional file 3.** Additional results. 3.1: Mortality data by information source and factors associated with capturing HDSS-registered births in EN-INDEPTH survey data. 3.2: Women’s recall of date-of-birth/death of their children in the EN-INDEPTH survey. 3.3: Number of deaths per 1000 live births- EN-INDEPTH study (blue) and HDSS data (red). 3.4: Number of women reporting ≥1children not living with them at the time of survey. 3.5: Cumulative mortality estimates among children born to women interviewed. 3.6: Distribution of mortality during the neonatal period. 3.7: Distribution of mortality during the neonatal period (excluding day 0-1) and first two years of life. 3.8A: Precision of survey estimates in capturing HDSS-recorded events – Bandim. 3.8B: Precision of survey estimates in capturing HDSS-recorded events – Dabat. 3.8C: Precision of survey estimates in capturing HDSS-recorded events – IgangaMayuge. 3.8D: Precision of survey estimates in capturing HDSS-recorded events – Matlab. 3.8E: Precision of survey estimates in capturing HDSS-recorded events – Kintampo. 3.9A: Precision of survey estimates in capturing HDSS-recorded births limiting to births followed in HDSS since date-of-birth– Bandim. 3.9B: Precision of survey estimates in capturing HDSS-recorded births limiting to births followed in HDSS since date-of-birth– Dabat. 3.9C: Precision of survey estimates in capturing HDSS-recorded births limiting to births followed in HDSS since date-of-birth– IgangaMayuge. 3.9D: Precision of survey estimates in capturing HDSS-recorded births limiting to births followed in HDSS since date-of-birth– Matlab. 3.9E: Precision of survey estimates in capturing HDSS-recorded births limiting to births followed in HDSS since date-of-birth– Kintampo. 3.10A: Precision of survey estimates in capturing HDSS-recorded events using wider age matching criteria - Bandim. 3.10B: Precision of survey estimates in capturing HDSS-recorded events using wider age matching criteria - Dabat. 3.10C: Precision of survey estimates in capturing HDSS-recorded events using wider age matching criteria – IgangaMayuge. 3.10D: Precision of survey estimates in capturing HDSS-recorded events using wider age matching criteria - Matlab. 3.10E: Precision of survey estimates in capturing HDSS-recorded events using wider age matching criteria - Kintampo. 3.11A: Precision of survey estimates in capturing HDSS-recorded events using wider age matching criteria - Bandim. 3.11B: Precision of survey estimates in capturing HDSS-recorded events using wider age matching criteria - Dabat. 3.11C: Precision of survey estimates in capturing HDSS-recorded events using wider age matching criteria – IgangaMayuge. 3.11D: Precision of survey estimates in capturing HDSS-recorded events using wider age matching criteria - Matlab. 3.11E: Precision of survey estimates in capturing HDSS-recorded events using wider age matching criteria – Kintampo.

**Additional file 4.** Ethical approval of local Institutional Review Boards.

## Data Availability

Data sharing and transfer agreements were jointly developed and signed by all collaborating partners. The datasets generated during the current study are deposited online at 10.17037/DATA.00001556 with data access subject to approval by collaborating parties.

## Abbreviations

DHS: Demographic and Health Surveys
ENAP: Every Newborn Action Plan
EN-INDEPTH: Every Newborn-INDEPTH
FBH+: Full Birth History (+ denotes additional questions on pregnancy losses)
FPH: Full pregnancy history
HDSS: Health and Demographic Surveillance System
ID: Identification number
LMICs: Low- and middle-income countries
MICS: Multiple Indicator Cluster Surveys
SDG: Sustainable Development Goals
WHO: World Health Organization
