# Supplementary material for: Neonatal and child mortality data in retrospective population-based surveys compared with prospective demographic surveillance: EN-INDEPTH study
Source: Popul Health Metr. 2021 Feb 8;19(Suppl 1):7. doi: 10.1186/s12963-020-00232-1 (PMC7869220; doi:10.1186/s12963-020-00232-1)
Supplement: Supplementary file 1 — Additional file 1. Additional methods. 1.1: Description of key differences between the HDSS sites. 1.2: Illustration of how observation time is counted in the EN-INDEPTH survey and different HDSS sites. 1.3: Additional methods for objective 2. [file 12963_2020_232_MOESM1_ESM.docx]

## **Additional file 1: Additional methods**

### **Additional file 1.1: Description of key differences between the HDSS sites**

| Site | Bandim (Guinea-Bissau) | Dabat (Ethiopia) | IgangaMayuge (Uganda) | Kintampo (Ghana) | Matlab (Bangladesh) |
| --- | --- | --- | --- | --- | --- |
| Population | ~200,000 | ~70,000 | ~80,000 | ~140,000 | ~250,000 |
| Frequency of pregnancy surveillance | Monthly in the urban area, bi-annually or bimonthly in the rural areas | Bi-annually | Bi-annually | Bi-annually | Bi-monthly |
| Frequency of follow up to children | Urban: Every 3-4 months (children<3), Rural: every 6 months (every 1-2 months, in some areas) | Bi-annually  + Community Key Informants monthly | Bi-annually + Community Key Informants monthly | 2011-13: Every 4 months  2014-15: Bi-annually 2016: Annually | Bi-monthly |
| HDSS definition of when a newborn is part of the HDSS | Date-of-birth if registered during pregnancy, otherwise date of first registration. | Date-of-birth if mother registered in HDSS, otherwise date of first registration. | Date-of-birth if mother registered in HDSS, otherwise date of first registration. | Date-of-birth if mother registered in HDSS, otherwise date of first registration. | Date-of-birth if mother registered in HDSS, otherwise date of first registration. |
| Sampling frame for survey | Women with a recorded birth outcome within last 5 years | Women of reproductive age | Women of reproductive age | Women with a recorded birth outcome within last 5 years | Women with a recorded birth outcome within last 5 years |

### **Additional file 1.2: Illustration of how observation time is counted in the EN-INDEPTH survey and different HDSS sites**


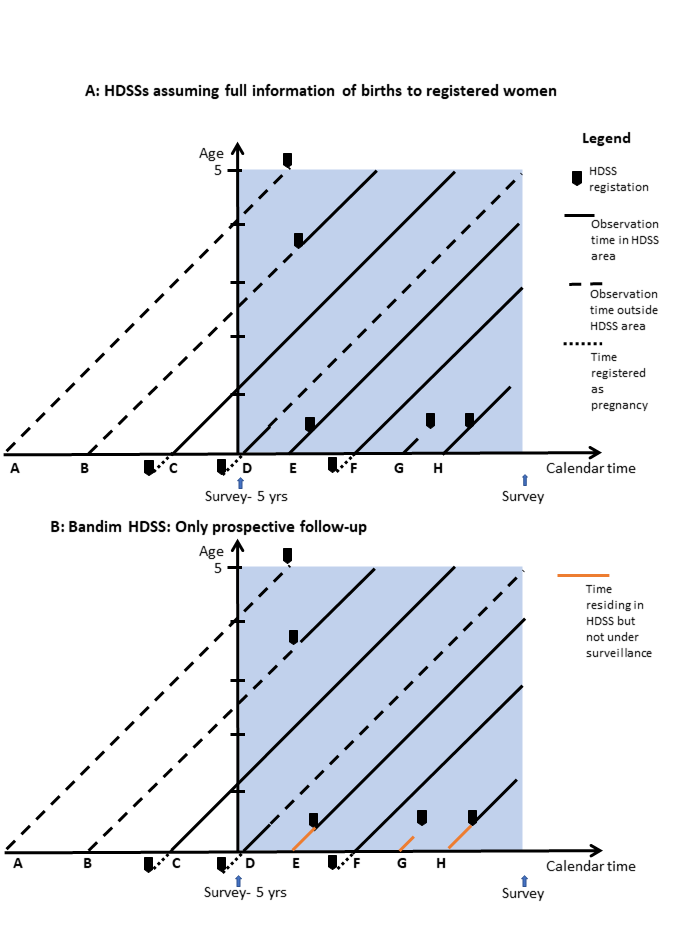


Note: The EN-INDEPTH survey counts all observation time of all children aged <5 years in the 5 years prior to the date of the survey (timelines in the blue rectangles). HDSS observation time is illustrated by full black lines. In four of the HDSS sites, full information on all births to registered women is assumed, and children therefore contribute survival time from birth if born to a registered woman, as shown in the top panel.

Children migrating with their mothers into the HDSS area (child A and B) contribute survival time from the date of registration in the HDSS if registered before 5 years of age (Child B). Children born in the HDSS area >5 years ago contribute time at risk from 5 years prior to the survey to 5 years of age (Child C). Children who have out-migrated from the HDSS contribute observation time until the date of migration (Child D). Children born to women registered in the area (Child E and F) contribute differently in Bandim and the other HDSSs if the pregnancy was not registered: In Bandim the child contributes observation time form the date of registration (Child E – at 6 months of age), while the other HDSSs count from the date of birth. Deaths in children born to registered women (Child G and H) also count differently by site. While Bandim considers a child at risk from date of registration until date of death (and therefore does not register child G), the other HDSS consider the child under risk from date of birth to the date of death.

Thus, in Bandim, early neonatal mortality estimates are based on a sample of all the births, which have occurred in the HDSS area. The rationale for using this approach is that Bandim assumes that a birth, which results in an early neonatal death (Child G) is less likely to be registered in the HDSS than a surviving child unless the woman is interviewed about the outcome of a registered pregnancy [1].

### **Additional file 1.3: Additional methods for objective 2**

*Kaplan-Meier estimates*: We describe the mortality rate during the past 5 years as one minus the survival probability estimated using Kaplan-Meier estimates, expressed as number of deaths by a particular age per 1000 live births.

*HDSS mortality estimates Dabat:* For the Kaplan-Meier estimates, observation time was censored at the date of EN-INDEPTH survey interview, except for Dabat HDSS where we censored at September 4, 2017. The reason for using a different censoring date, was that Dabat had changed data collection methodology, and therefore data from the most recent rounds were not linked to the prior data prior to initiating the analyses. Hence, we applied the date of censoring corresponding to the last round of linked data.

*Distribution of mortality:* To identify factors associated with heaping, we produce separate graphs based on data stratified by survey factors (site, period of recall (within the past 2 years vs before)), maternal factors (parity, educational status, socioeconomic status) and child factors (sex).

*Heaping index:* We quantified heaping by calculating a heaping index as described by Hill and Chow [2]. The index describes how many times the number of deaths on day 7 was higher than the average daily number of deaths between day 5 to 9. Similarly, we calculated a heaping index for 12 months as the number reported at 12 months / average number of monthly deaths between 10 and 14 months.

**References**

1. Thysen SM, Fernandes M, Benn CS, Aaby P, Fisker AB: **Cohort profile : Bandim Health Project's (BHP) rural Health and Demographic Surveillance System (HDSS)-a nationally representative HDSS in Guinea-Bissau.** *BMJ Open* 2019, **9:**e028775.

2. Hill K, Choi Y: **Neonatal mortality in the developing world.** *Demographic Research* 2006, **14:**429-452.
