# Supplementary material for: Neonatal and child mortality data in retrospective population-based surveys compared with prospective demographic surveillance: EN-INDEPTH study
Source: Popul Health Metr. 2021 Feb 8;19(Suppl 1):7. doi: 10.1186/s12963-020-00232-1 (PMC7869220; doi:10.1186/s12963-020-00232-1)
Supplement: Supplementary file 3 — Additional file 3. Additional results. 3.1: Mortality data by information source and factors associated with capturing HDSS-registered births in EN-INDEPTH survey data. 3.2: Women’s recall of date-of-birth/death of their children in the EN-INDEPTH survey. 3.3: Number of deaths per 1000 live births- EN-INDEPTH study (blue) and HDSS data (red). 3.4: Number of women reporting ≥1children not living with them at the time of survey. 3.5: Cumulative mortality estimates among children born to women interviewed. 3.6: Distribution of mortality during the neonatal period. 3.7: Distribution of mortality during the neonatal period (excluding day 0-1) and first two years of life. 3.8A: Precision of survey estimates in capturing HDSS-recorded events – Bandim. 3.8B: Precision of survey estimates in capturing HDSS-recorded events – Dabat. 3.8C: Precision of survey estimates in capturing HDSS-recorded events – IgangaMayuge. 3.8D: Precision of survey estimates in capturing HDSS-recorded events – Matlab. 3.8E: Precision of survey estimates in capturing HDSS-recorded events – Kintampo. 3.9A: Precision of survey estimates in capturing HDSS-recorded births limiting to births followed in HDSS since date-of-birth– Bandim. 3.9B: Precision of survey estimates in capturing HDSS-recorded births limiting to births followed in HDSS since date-of-birth– Dabat. 3.9C: Precision of survey estimates in capturing HDSS-recorded births limiting to births followed in HDSS since date-of-birth– IgangaMayuge. 3.9D: Precision of survey estimates in capturing HDSS-recorded births limiting to births followed in HDSS since date-of-birth– Matlab. 3.9E: Precision of survey estimates in capturing HDSS-recorded births limiting to births followed in HDSS since date-of-birth– Kintampo. 3.10A: Precision of survey estimates in capturing HDSS-recorded events using wider age matching criteria - Bandim. 3.10B: Precision of survey estimates in capturing HDSS-recorded events using wider age matching criteria - Dabat. 3.10C: [file 12963_2020_232_MOESM3_ESM.docx]

## **Additional file 3: Additional results**

### **Additional file 3.1: Mortality data by information source and factors associated with capturing HDSS-registered births in EN-INDEPTH survey data**

**Mortality data by source of information**

The proportion of women interviewed in the EN-INDEPTH survey who reported live births contributing survival information during the 5 years prior to the interview was higher where the sampling was among women with a registered birth during the past 5 years (97-99%) than in the sites where interviews were conducted among all women of reproductive age (84 and 87%). The subset of interviewed women who also had children under-5 under HDSS surveillance was lower (97% in Bandim, 95% in Matlab, 96% in Kintampo, 84% in IgangaMayuge and 55% in Dabat where the HDSS data was not complete up to the time of the survey) (Table 2).

While the number of children under surveillance during the five years prior to the survey was similar for HDSS and EN-INDEPTH survey data in Matlab (31,992 vs 32,308), the numbers in the comparable period in the HDSS data were markedly lower in the other HDSS sites: 23% lower in IgangaMayuge, 12% in Bandim and 44% in Kintampo (Table 2). When comparing the number of deaths for children during the past 5 years born to the interviewed women, the numbers were similar for Matlab (an additional 7% deaths recorded in the EN-INDEPTH survey) and for Bandim HDSS (an additional 2% in the EN-INDEPTH survey), while Iganga and Kintampo had an additional 26% and 45% in the EN-INDEPTH survey. While higher numbers of deaths in the EN-INDEPTH survey than in the HDSS data were observed for both neonatal, infant and under-5 deaths in the other sites, the data from Bandim indicated that more deaths in the infant and neonatal period were captured through the HDSS than through the EN-INDEPTH survey (Table 2).

Based on the EN-INDEPTH survey data, the neonatal mortality was 43% of under-5 mortality in Kintampo, 48% in IgangaMayuge, 54% in Bandim, 55% in Dabat, and 61% in Matlab. The share of under-5 mortality made up by neonatal mortality estimated through the HDSS was 42% in Dabat, 44% in IgangaMayuge and Kintampo, 57% in Matlab and 63% in Bandim.

**Factors associated with capturing an HDSS registered birth in the EN-INDEPTH survey data**

In all sites, longer recall was associated with lower matching probability, but the magnitude differed widely by site from a 42% lover matching probability in IgangaMayuge if the birth was more than 2 years ago to a 7% lower matching probability in Kintampo (Figure 6, Supplementary file 3.9A-E).

Limiting the attempted matched population to children followed in the HDSS data since birth, increased the proportion matched to 87% in Bandim, 63% in Dabat, 53% in IgangaMayuge, 90% in Matlab and 90% in Kintampo (Supplementary file 3.10A-E). Relaxing the matching criteria to HDSS birthdate +/-3 months increased the proportion matched to 85%, 63%, 58%, 91% and 90% in the five sites (Supplementary File 3.11A-E). Further relaxing the matching criteria to +/-9 months increased the proportions to 90%, 68%, 71%, 93% and 93% in the five sites (Supplementary File 3.12A-E). The sensitivity analyses identified the same patterns of background factors associated with matching (Supplementary Files 3.9A-E, 3.10A-E, 3.11A-E and 3.12A-E).

### **Additional file 3.2: Women’s recall of date-of-birth / death of their children in the EN-INDEPTH survey**

|  | **Bandim**  **n(%)** | **Dabat**  **n(%)** | **IgangaMayuge n(%)** | **Matlab n(%)** | **Kintampo n(%)** |
| --- | --- | --- | --- | --- | --- |
| Number of women reporting children | 9,391 | 8,963 | 8,106 | 21,035 | 12,143 |
| **Children born to women interviewed** | | | | | |
| All | 28,043 | 36,920 | 38,361 | 45,612 | 46,085 |
| Number of children per interviewed woman. Median (IQR) | 3 (1-4) | 4 (2-6) | 4 (2-7) | 2 (1-3) | 3 (2-5) |
| Last 5 years | 18,423 | 16,029 | 16,044 | 32,308 | 27,013 |
| **Recorded date-of-birth – All** | | | | | |
| Day recorded | 24,063 (86) | 36,092 (98) | 33,646 (88) | 42,979 (94) | 36,921 (80) |
| Only month recorded | 1,455 (5) | 615 (2) | 2,115 (6) | 1,951 (4) | 1,647 (4) |
| Only year recorded | 2,438 (9) | 213 (1) | 2,600 (7) | 680 (1) | 7,517 (16) |
| No date recorded | 87 (0) | 0 (0) | 0 (0) | 2 (0) | 0 (0) |
| **Recorded date-of-birth - Last 5 years** | | | | | |
| Day recorded | 17,047 (93) | 15,883 (99) | 15,034 (94) | 31,553 (98) | 25,181 (93) |
| Only month recorded | 559 (3) | 117 (1) | 493 (3) | 607 (2) | 455 (2) |
| Only year recorded | 754 (4) | 29 (0) | 517 (3) | 147 (0) | 1377 (5) |
| No date recorded | 63 (0) | 0 (0) | 0 (0) | 1 (0) | 0 (0) |
| **Deaths among under 5 children born to interviewed women** | | | | | |
| All deaths | 2752 | 2785 | 3083 | 2187 | 3369 |
| Deaths during the last 5 years | 766 | 390 | 477 | 717 | 714 |
| Last 5 years, interviewed with FPH | 373 | 186 | 229 | 373 | 346 |
| **Recorded date of death - Last 5 years (among children born to women interviewed with FPH^1^)** | | | | | |
| Day recorded | 192 (51) | 175 (94) | 144 (63) | 306 (82) | 103 (30) |
| Only month recorded | 101 (27) | 10 (5) | 52 (23) | 48 (13) | 89 (26) |
| Only year recorded | 80 (21) | 1 (1) | 33 (14) | 19 (5) | 154 (45) |

1: Date of death was only collected in the Full Pregnancy History (FPH) and not as part of the Full Birth History (FBH+).

### **Additional file 3.3: Number of deaths per 1000 live births^1^- EN-INDEPTH study (blue) and HDSS data^2^ (red)**


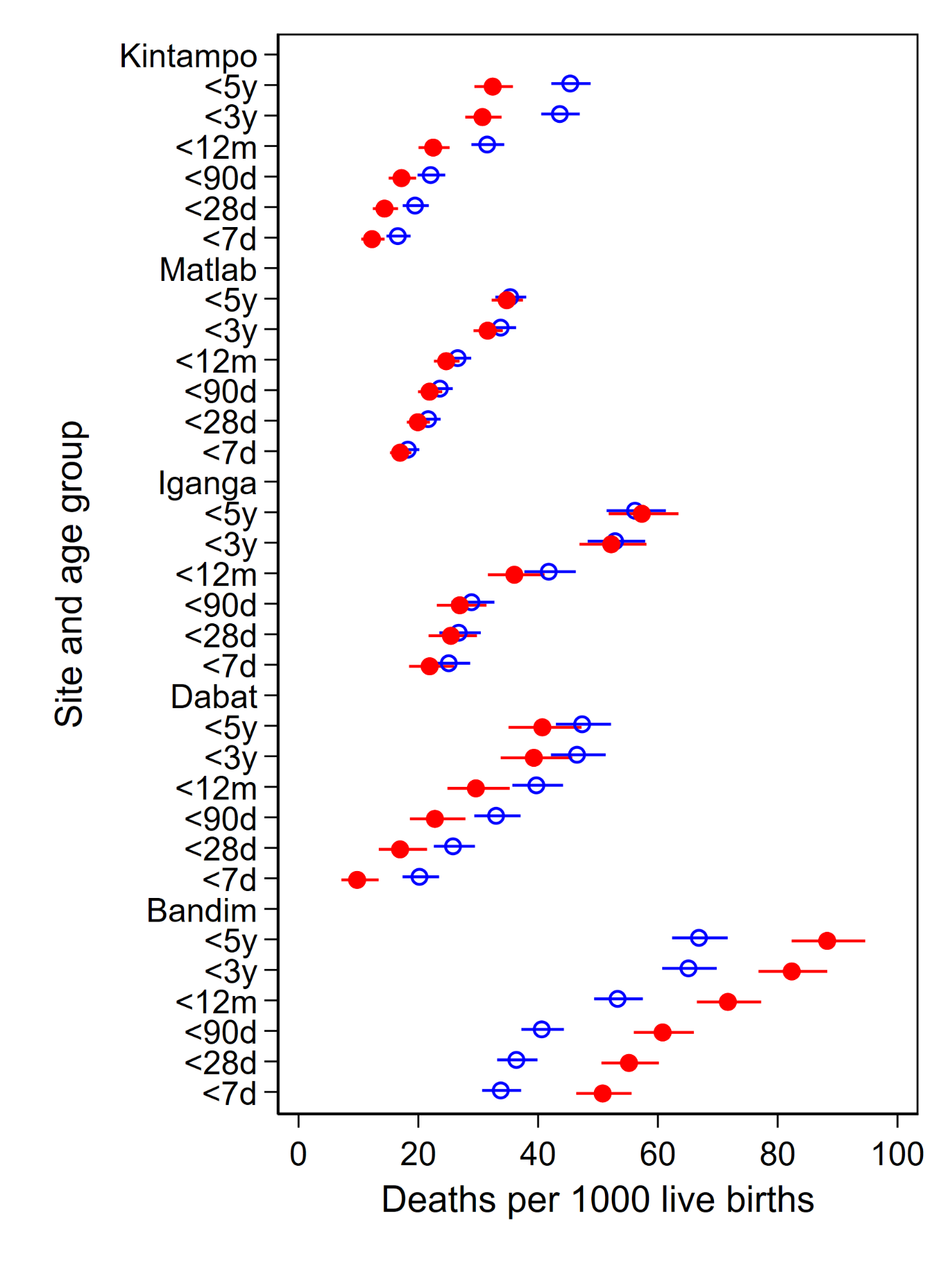


*1: During the 5 years preceding the survey interview*

*2: Among children registered to the same women*

*Note: Estimated using Kaplan Meier failure estimates.*

###

### **Additional file 3.4: Number of women reporting ≥1 children not living with them at the time of survey**

|  | Bandim (Guinea-Bissau) | Dabat (Ethiopia) | IgangaMayuge (Uganda) | Matlab (Bangladesh) | Kintampo (Ghana) |
| --- | --- | --- | --- | --- | --- |
| Number of women with children contributing survival information in the past 5 years | 9,319 | 7,781 | 6,901 | 20,484 | 12,083 |
| Reporting any child living elsewhere | 2487 (27%) | 2208 (28%) | 1894 (27%) | 1894 (9%) | 3880 (32%) |
| Number of women with no children not contributing survival information in the past 5 years | 5,182 | 2,862 | 3,021 | 13,300 | 5,300 |
| Reporting any child living elsewhere | 624 (12%) | 108 (4%) | 211 (7%) | 319 (2%) | 615 (12%) |

### **Additional file 3.5: Cumulative mortality estimates among children born to women interviewed^1^**

**
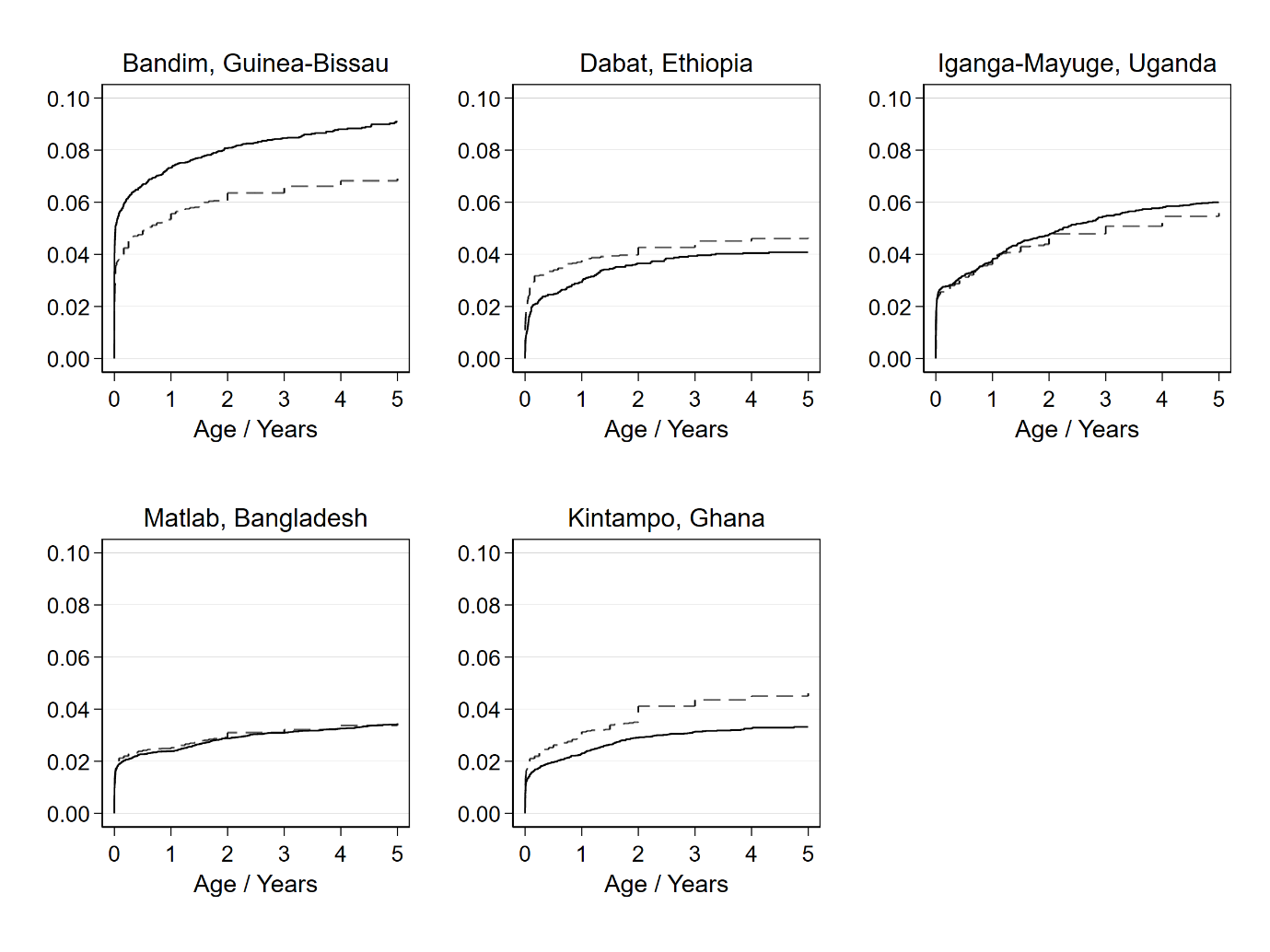
**

*1: Limiting the analysis to women resident in same city/village/town during the last 5 years*

**Additional file 3.6: Distribution of mortality during the neonatal period^1^**
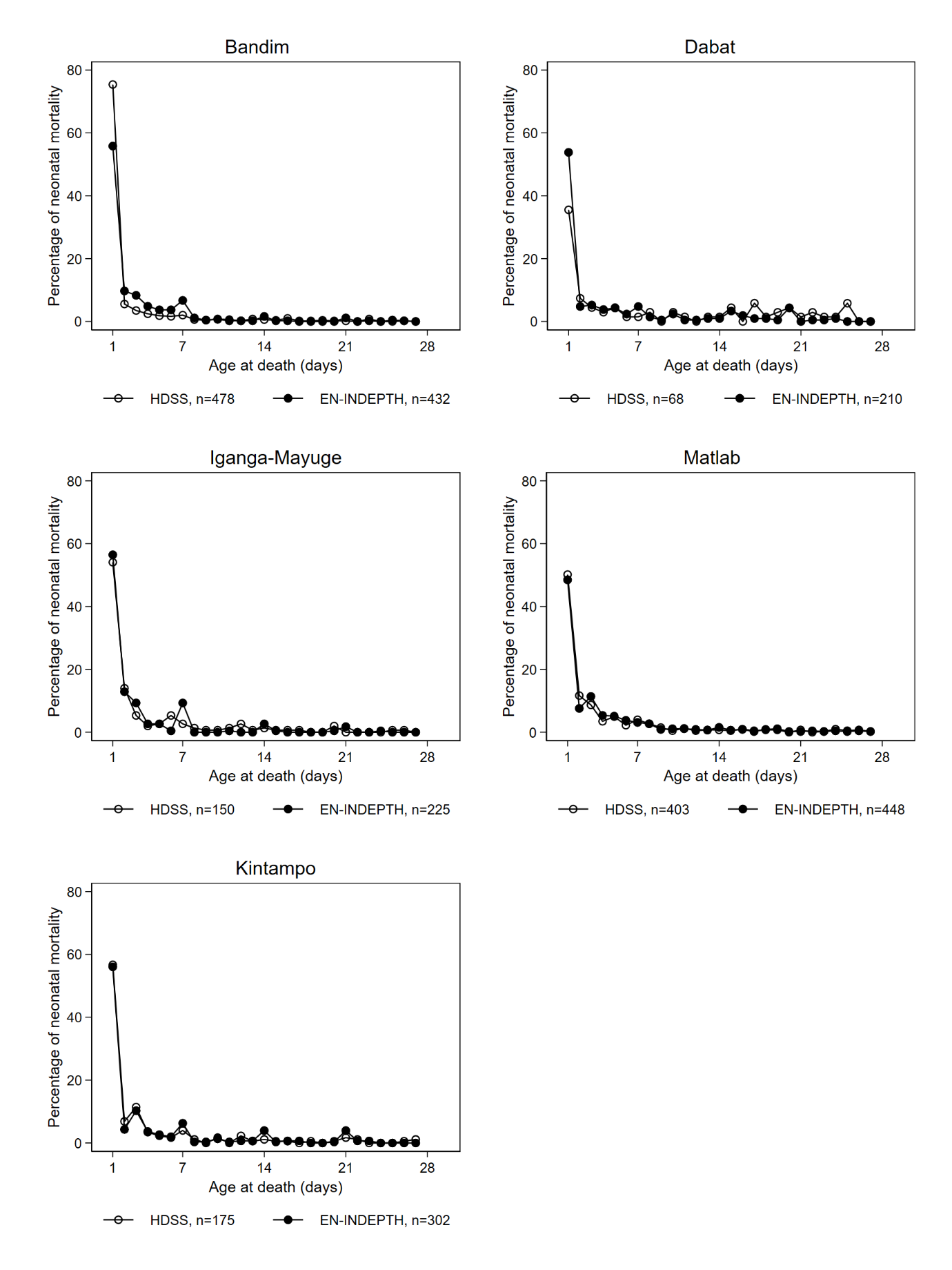


*1: Deaths on day 0 and 1 appear on all depicted on day 1. Percentages of neonatal deaths by day*

### **Additional file 3.7: Distribution of mortality during the neonatal period (excluding day 0-1) and first two years of life^1^**


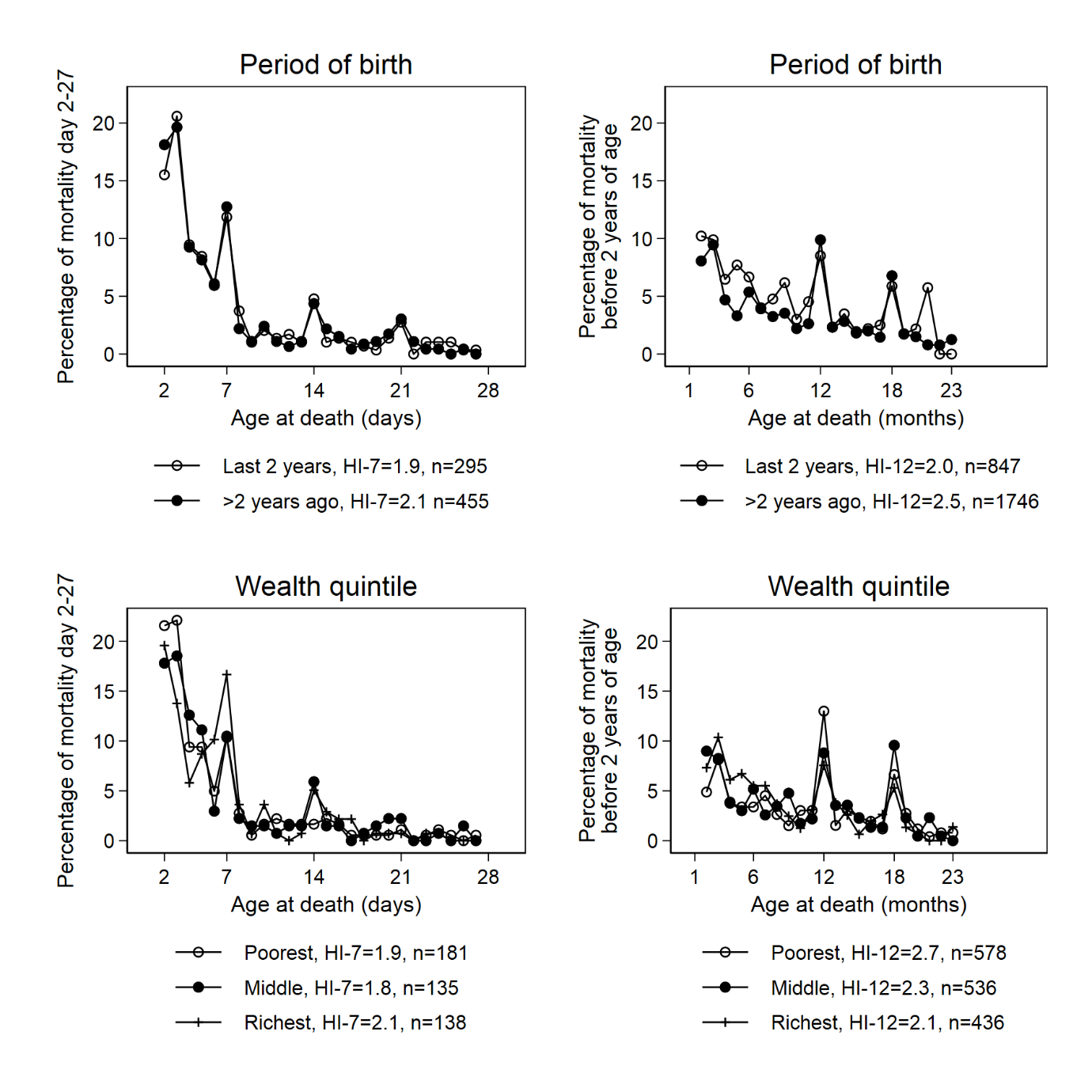
*1: Percentages of child deaths by day/month of death. HI-7: Heaping index for deaths on day 7, HI-12: Heaping index for death at 12 months*

### **Additional file 3.8A: Precision of survey estimates in capturing HDSS recorded events – Bandim**

|  |  | Number of children born to interviewed women under HDSS surveillance during the five years prior to EN-INDEPTH survey | Number of births (%) matching (+/-1 months) to a survey recorded birth | RR of capture in the survey (95%CI) |
| --- | --- | --- | --- | --- |
|  | All | 16191 | 13845 (86) |  |
| Recall period | |  |  |  |
|  | 0-1 years | 4887 | 4598 (94) | 1 (ref) |
|  | >2 years | 11304 | 9247 (82) | 0.87 (0.86-0.88) |
| **Survey tool** | | | | |
|  | Birth history | 8284 | 7120 (86) | 1 (ref) |
|  | Pregnancy history | 7907 | 6725 (85) | 0.99 (0.98-1.00) |
| **Child factors** | | | | |
| Survival status in HDSS | |  |  |  |
|  | Alive | 15438 | 13496 (87) | 1 (ref) |
|  | Dead before 5 years | 753 | 349 (46) | 0.53 (0.49-0.57) |
|  | Alive | 15438 | 13496 (87) | 1 (ref) |
|  | Dead day 0-6 | 431 | 174 (40) | 0.46 (0.41-0.52) |
|  | Dead day 7-27 | 48 | 26 (54) | 0.62 (0.47-0.81) |
|  | Dead day 28-364 | 176 | 108 (61) | 0.70 (0.62-0.79) |
|  | Dead 1-2 years | 112 | 68 (61) | 0.69 (0.60-0.81) |
|  | Dead 3-4 years | 28 | 10 (36) | 0.41 (0.25-0.67) |
| Sex | |  |  |  |
|  | Male | 8281 | 7108 (86) | 1 (ref) |
|  | Female | 7893 | 6734 (85) | 0.99 (0.98-1.01) |
|  | Missing | 17 | 3 (18) |  |
| **Maternal factors** | | | | |
| Education | |  |  |  |
|  | None | 5588 | 4488 (80) | 1 (ref) |
|  | Primary school | 4767 | 4083 (86) | 1.07 (1.05-1.09) |
|  | Secondary school | 4758 | 4360 (92) | 1.14 (1.12-1.16) |
|  | Higher education | 967 | 913 (94) | 1.18 (1.15-1.20) |
|  | Missing | 111 | 1 (1) |  |
| Parity | |  |  |  |
|  | 1 | 2415 | 2257 (93) | 1 (ref) |
|  | 2 | 3255 | 2909 (89) | 0.96 (0.94-0.97) |
|  | 3 | 3171 | 2776 (88) | 0.94 (0.92-0.95) |
|  | 4 | 2596 | 2150 (83) | 0.89 (0.87-0.91) |
|  | 5+ | 4644 | 3753 (81) | 0.86 (0.85-0.88) |
|  | Missing | 110 | 0 (0) |  |
| Wealth quintiles | |  |  |  |
|  | Poorest | 2898 | 2610 (90) | 1 (ref) |
|  | 2 | 2961 | 2620 (88) | 0.98 (0.96-1.00) |
|  | 3 | 3002 | 2582 (86) | 0.96 (0.94-0.98) |
|  | 4 | 3138 | 2688 (86) | 0.95 (0.93-0.97) |
|  | Richest | 4082 | 3345 (82) | 0.91 (0.89-0.93) |
|  | Missing | 110 | 0 (0) |  |

### **Additional file 3.8B: Precision of survey estimates in capturing HDSS recorded events – Dabat**

|  |  | Number of children born to interviewed women under HDSS surveillance during the five years prior to EN-INDEPTH survey | Number of births (%) matching (+/-1 months) to a survey recorded birth | RR of capture in the survey (95%CI) |
| --- | --- | --- | --- | --- |
|  | All | 7999 | 4664 (58) |  |
| Recall period | |  |  |  |
|  | 0-1 years | 996 | 731 (73) | 1 (ref) |
|  | >2 years | 7003 | 3933 (56) | 0.77 (0.73-0.80) |
| **Survey tool** | | | | |
|  | Birth history | 3976 | 2380 (60) | 1 (ref) |
|  | Pregnancy history | 3928 | 2284 (58) | 0.97 (0.93-1.01) |
| **Child factors** | | | | |
| Survival status in HDSS | |  |  |  |
|  | Alive | 7833 | 4609 (59) | 1 (ref) |
|  | Dead before 5 years | 166 | 55 (33) | 0.56 (0.45-0.71) |
|  | Alive | 7833 | 4609 (59) | 1 (ref) |
|  | Dead day 0-6 | 38 | 14 (37) | 0.63 (0.41-0.97) |
|  | Dead day 7-27 | 30 | 8 (27) | 0.45 (0.25-0.83) |
|  | Dead day 28-364 | 54 | 15 (28) | 0.47 (0.30-0.74) |
|  | Dead 1-2 years | 39 | 16 (41) | 0.70 (0.48-1.02) |
|  | Dead 3-4 years | 5 | 2 (40) | 0.68 (0.23-1.99) |
| Sex | |  |  |  |
|  | Male | 2716 | 1777 (65) | 1 (ref) |
|  | Female | 2706 | 1774 (66) | 1.00 (0.96-1.04) |
|  | Missing | 2577 | 1113 (43) |  |
| **Maternal factors** | | | | |
| Education | |  |  |  |
|  | None | 5497 | 2901 (53) | 1 (ref) |
|  | Primary school | 1571 | 1026 (65) | 1.24 (1.18-1.30) |
|  | Secondary school | 454 | 348 (77) | 1.45 (1.37-1.54) |
|  | Higher education | 477 | 389 (82) | 1.55 (1.47-1.63) |
|  | Missing | 0 |  |  |
| Parity | |  |  |  |
|  | 1 | 534 | 360 (67) | 1 (ref) |
|  | 2 | 980 | 709 (72) | 1.07 (1.00-1.16) |
|  | 3 | 1125 | 770 (68) | 1.02 (0.94-1.09) |
|  | 4 | 1250 | 754 (60) | 0.89 (0.83-0.97) |
|  | 5+ | 4015 | 2071 (52) | 0.77 (0.71-0.82) |
|  | Missing | 95 | 0 (0) |  |
| Wealth quintiles | |  |  |  |
|  | Poorest | 2453 | 1262 (51) | 1 (ref) |
|  | 2 | 1508 | 857 (57) | 1.10 (1.04-1.18) |
|  | 3 | 1634 | 945 (58) | 1.12 (1.06-1.20) |
|  | 4 | 1343 | 844 (63) | 1.22 (1.15-1.30) |
|  | Richest | 966 | 756 (78) | 1.52 (1.44-1.61) |
|  | Missing | 95 | 0 (0) |  |

### **Additional file 3.8C: Precision of survey estimates in capturing HDSS recorded events – IgangaMayuge**

|  |  | Number of children born to interviewed women under HDSS surveillance during the five years prior to EN-INDEPTH survey | Number of births (%) matching (+/-1 months) to a survey recorded birth | RR of capture in the survey (95%CI) |
| --- | --- | --- | --- | --- |
|  | All | 12401 | 6278 (51) |  |
| Recall period | |  |  |  |
|  | 0-1 years | 2508 | 1903 (76) | 1 (ref) |
|  | >2 years | 9893 | 4375 (44) | 0.58 (0.56-0.60) |
| **Survey tool** | | | | |
|  | Birth history | 6259 | 3119 (50) | 1 (ref) |
|  | Pregnancy history | 6142 | 3159 (51) | 1.03 (0.99-1.08) |
| **Child factors** | | | | |
| Survival status in HDSS | |  |  |  |
|  | Alive | 12049 | 6176 (51) | 1 (ref) |
|  | Dead before 5 years | 352 | 102 (29) | 0.57 (0.47-0.67) |
|  | Alive | 12049 | 6176 (51) | 1 (ref) |
|  | Dead day 0-6 | 125 | 35 (28) | 0.55 (0.40-0.75) |
|  | Dead day 7-27 | 25 | 11 (44) | 0.86 (0.53-1.38) |
|  | Dead day 28-364 | 66 | 28 (42) | 0.83 (0.62-1.11) |
|  | Dead 1-2 years | 104 | 22 (21) | 0.41 (0.28-0.60) |
|  | Dead 3-4 years | 34 | 8 (24) | 0.46 (0.25-0.84) |
| Sex | |  |  |  |
|  | Male | 6276 | 3199 (51) | 1 (ref) |
|  | Female | 6125 | 3079 (50) | 0.99 (0.95-1.02) |
|  | Missing | 0 |  |  |
| **Maternal factors** | | | | |
| Education | |  |  |  |
|  | None | 1312 | 522 (40) | 1 (ref) |
|  | Primary school | 7002 | 3473 (50) | 1.25 (1.15-1.35) |
|  | Secondary school | 3554 | 1970 (55) | 1.39 (1.28-1.51) |
|  | Higher education | 533 | 313 (59) | 1.48 (1.32-1.66) |
|  | Missing | 0 |  |  |
| Parity | |  |  |  |
|  | 1 | 712 | 454 (64) | 1 (ref) |
|  | 2 | 1222 | 782 (64) | 1.00 (0.93-1.08) |
|  | 3 | 1509 | 979 (65) | 1.02 (0.95-1.10) |
|  | 4 | 1668 | 979 (59) | 0.92 (0.85-0.99) |
|  | 5+ | 6966 | 3084 (44) | 0.69 (0.65-0.74) |
|  | Missing | 324 | 0 (0) |  |
| Wealth quintiles | |  |  |  |
|  | Poorest | 3438 | 1767 (51) | 1 (ref) |
|  | 2 | 2819 | 1436 (51) | 0.99 (0.93-1.05) |
|  | 3 | 2289 | 1228 (54) | 1.04 (0.98-1.11) |
|  | 4 | 1941 | 999 (51) | 1.00 (0.94-1.07) |
|  | Richest | 1590 | 848 (53) | 1.04 (0.97-1.11) |
|  | Missing | 324 | 0 (0) |  |

### **Additional file 3.8D: Precision of survey estimates in capturing HDSS recorded events – Matlab**

|  |  | Number of children born to interviewed women under HDSS surveillance during the five years prior to EN-INDEPTH survey | Number of births (%) matching (+/-1 months) to a survey recorded birth | RR of capture in the survey (95%CI) |
| --- | --- | --- | --- | --- |
|  | All | 31992 | 28526 (89) |  |
| Recall period | |  |  |  |
|  | 0-1 years | 8240 | 7928 (96) | 1 (ref) |
|  | >2 years | 23752 | 20598 (87) | 0.90 (0.90-0.91) |
| **Survey tool** | | | | |
|  | Birth history | 16122 | 14434 (90) | 1 (ref) |
|  | Pregnancy history | 15799 | 14092 (89) | 1.00 (0.99-1.00) |
| **Child factors** | | | | |
| Survival status in HDSS | |  |  |  |
|  | Alive | 31324 | 28053 (90) | 1 (ref) |
|  | Dead before 5 years | 668 | 473 (71) | 0.79 (0.75-0.83) |
|  | Alive | 31324 | 28053 (90) | 1 (ref) |
|  | Dead day 0-6 | 327 | 225 (69) | 0.77 (0.71-0.83) |
|  | Dead day 7-27 | 76 | 55 (72) | 0.81 (0.70-0.93) |
|  | Dead day 28-364 | 108 | 85 (79) | 0.88 (0.80-0.97) |
|  | Dead 1-2 years | 141 | 101 (72) | 0.80 (0.72-0.89) |
|  | Dead 3-4 years | 51 | 39 (76) | 0.85 (0.73-0.99) |
| Sex | |  |  |  |
|  | Male | 15935 | 14311 (90) | 1 (ref) |
|  | Female | 16057 | 14215 (89) | 0.99 (0.98-0.99) |
|  | Missing | 0 |  |  |
| **Maternal factors** | | | | |
| Education | |  |  |  |
|  | None | 1385 | 1138 (82) | 1 (ref) |
|  | Primary school | 6248 | 5293 (85) | 1.03 (1.00-1.06) |
|  | Secondary school | 20510 | 18494 (90) | 1.10 (1.07-1.13) |
|  | Higher education | 3848 | 3601 (94) | 1.14 (1.11-1.17) |
|  | Missing | 1 | 0 (0) |  |
| Parity | |  |  |  |
|  | 1 | 6330 | 6002 (95) | 1 (ref) |
|  | 2 | 12909 | 11683 (91) | 0.95 (0.95-0.96) |
|  | 3 | 8353 | 7293 (87) | 0.92 (0.91-0.93) |
|  | 4 | 3092 | 2553 (83) | 0.87 (0.85-0.89) |
|  | 5+ | 1237 | 995 (80) | 0.85 (0.82-0.87) |
|  | Missing | 71 | 0 (0) |  |
| Wealth quintiles | |  |  |  |
|  | Poorest | 6763 | 5850 (87) | 1 (ref) |
|  | 2 | 6405 | 5677 (89) | 1.02 (1.01-1.04) |
|  | 3 | 6293 | 5630 (89) | 1.03 (1.02-1.05) |
|  | 4 | 6212 | 5641 (91) | 1.05 (1.04-1.06) |
|  | Richest | 6248 | 5728 (92) | 1.06 (1.05-1.07) |
|  | Missing | 71 | 0 (0) |  |

### **Additional file 3.8E: Precision of survey estimates in capturing HDSS recorded events – Kintampo**

|  |  | Number of children born to interviewed women under HDSS surveillance during the five years prior to EN-INDEPTH survey | Number of births (%) matching (+/-1 months) to a survey recorded birth | RR of capture in the survey (95%CI) |
| --- | --- | --- | --- | --- |
|  | All | 15185 | 13312 (88) |  |
| Recall period | |  |  |  |
|  | 0-1 years | 3399 | 3159 (93) | 1 (ref) |
|  | >2 years | 11786 | 10153 (86) | 0.93 (0.92-0.94) |
| **Survey tool** | | | | |
|  | Birth history | 7535 | 6638 (88) | 1 (ref) |
|  | Pregnancy history | 7650 | 6674 (87) | 0.99 (0.98-1.00) |
| **Child factors** | | | | |
| Survival status in HDSS | |  |  |  |
|  | Alive | 14799 | 13106 (89) | 1 (ref) |
|  | Dead before 5 years | 386 | 206 (53) | 0.60 (0.55-0.66) |
|  | Alive | 14799 | 13106 (89) | 1 (ref) |
|  | Dead day 0-6 | 144 | 66 (46) | 0.52 (0.43-0.62) |
|  | Dead day 7-27 | 31 | 23 (74) | 0.84 (0.68-1.03) |
|  | Dead day 28-364 | 110 | 58 (53) | 0.60 (0.50-0.71) |
|  | Dead 1-2 years | 100 | 58 (58) | 0.65 (0.55-0.77) |
|  | Dead 3-4 years | 11 | 6 (55) | 0.62 (0.36-1.06) |
| Sex | |  |  |  |
|  | Male | 7790 | 6863 (88) | 1 (ref) |
|  | Female | 7395 | 6449 (87) | 0.99 (0.98-1.00) |
|  | Missing | 0 |  |  |
| **Maternal factors** | | | | |
| Education | |  |  |  |
|  | None | 6183 | 5411 (88) | 1 (ref) |
|  | Primary school | 8363 | 7377 (88) | 1.01 (0.99-1.02) |
|  | Secondary school | 478 | 394 (82) | 0.94 (0.90-0.99) |
|  | Higher education | 161 | 130 (81) | 0.92 (0.85-1.00) |
|  | Missing | 0 |  |  |
| Parity | |  |  |  |
|  | 1 | 1930 | 1594 (83) | 1 (ref) |
|  | 2 | 2564 | 2243 (87) | 1.06 (1.03-1.09) |
|  | 3 | 2647 | 2370 (90) | 1.08 (1.06-1.11) |
|  | 4 | 2384 | 2112 (89) | 1.07 (1.04-1.10) |
|  | 5+ | 5658 | 4993 (88) | 1.07 (1.04-1.09) |
|  | Missing | 2 | 0 (0) |  |
| Wealth quintiles | |  |  |  |
|  | Poorest | 3180 | 2781 (87) | 1 (ref) |
|  | 2 | 3064 | 2692 (88) | 1.00 (0.99-1.02) |
|  | 3 | 3020 | 2677 (89) | 1.01 (0.99-1.03) |
|  | 4 | 2969 | 2615 (88) | 1.01 (0.99-1.03) |
|  | Richest | 2950 | 2547 (86) | 0.99 (0.97-1.01) |
|  | Missing | 2 | 0 (0) |  |

### **Additional file 3.9A: Precision of survey estimates in capturing HDSS-recorded births limiting to births followed in HDSS since date-of-birth– Bandim**

|  |  | Number of children born to interviewed women under HDSS surveillance during the five years prior to EN-INDEPTH survey | Number of births (%) matching (+/-1 months) to a survey recorded birth | RR of capture in the survey (95%CI) |
| --- | --- | --- | --- | --- |
|  | All | 11834 | 10204 (86) |  |
| Recall period | |  |  |  |
|  | 0-1 years | 3935 | 3699 (94) | 1 (ref) |
|  | >2 years | 7899 | 6505 (82) | 0.88 (0.86-0.89) |
| **Survey tool** | | | | |
|  | Birth history | 6082 | 5270 (87) | 1 (ref) |
|  | Preganancy history | 5752 | 4934 (86) | 0.99 (0.97-1.01) |
| **Child factors** | | | | |
| Survival status in HDSS | |  |  |  |
|  | Alive | 11139 | 9885 (89) | 1 (ref) |
|  | Dead before 5 years | 695 | 319 (46) | 0.99 (0.97-1.01) |
|  | Alive | 11139 | 9885 (89) | 1 (ref) |
|  | Dead day 0-6 | 428 | 172 (40) | 0.45 (0.40-0.51) |
|  | Dead day 7-27 | 44 | 24 (55) | 0.61 (0.47-0.81) |
|  | Dead day 28-364 | 149 | 93 (62) | 0.70 (0.62-0.80) |
|  | Dead 1-2 years | 86 | 52 (60) | 0.68 (0.57-0.81) |
|  | Dead 3-4 years | 18 | 6 (33) | 0.38 (0.20-0.72) |
| Sex | |  |  |  |
|  | Male | 6040 | 5237 (87) | 1 (ref) |
|  | Female | 5777 | 4964 (86) | 0.99 (0.98-1.01) |
|  | Missing | 17 | 3 (18) |  |
| **Maternal factors** | | | | |
| Education | |  |  |  |
|  | None | 4113 | 3357 (82) | 1 (ref) |
|  | Primary school | 3478 | 2999 (86) | 1.06 (1.03-1.08) |
|  | Secondary school | 3521 | 3238 (92) | 1.13 (1.11-1.15) |
|  | Higher education | 645 | 609 (94) | 1.16 (1.13-1.19) |
|  | Missing | 77 | 1 (1) |  |
| Parity | |  |  |  |
|  | 1 | 1722 | 1608 (93) | 1 (ref) |
|  | 2 | 2304 | 2066 (90) | 0.96 (0.94-0.98) |
|  | 3 | 2301 | 2038 (89) | 0.95 (0.93-0.97) |
|  | 4 | 1931 | 1618 (84) | 0.90 (0.87-0.92) |
|  | 5+ | 3500 | 2874 (82) | 0.88 (0.86-0.90) |
|  | Missing | 76 | 0 (0) |  |
| Wealth quintiles | |  |  |  |
|  | Poorest | 2113 | 1915 (91) | 1 (ref) |
|  | 2 | 2220 | 1969 (89) | 0.98 (0.96-1.00) |
|  | 3 | 2291 | 1981 (86) | 0.95 (0.93-0.98) |
|  | 4 | 2319 | 1994 (86) | 0.95 (0.93-0.97) |
|  | Richest | 2815 | 2345 (83) | 0.92 (0.90-0.94) |
|  | Missing | 76 | 0 (0) |  |

### **Additional file 3.9B: Precision of survey estimates in capturing HDSS-recorded births limiting to births followed in HDSS since date-of-birth– Dabat**

|  |  | Number of children born to interviewed women under HDSS surveillance during the five years prior to EN-INDEPTH survey | Number of births (%) matching (+/-1 months) to a survey recorded birth | RR of capture in the survey (95%CI) |
| --- | --- | --- | --- | --- |
|  | All | 7247 | 4205 (58) |  |
| Recall period | |  |  |  |
|  | 0-1 years | 994 | 729 (73) | 1 (ref) |
|  | >2 years | 6253 | 3476 (56) | 0.76 (0.72-0.79) |
| **Survey tool** | | | | |
|  | Birth history | 3617 | 2155 (60) | 1 (ref) |
|  | Pregnancy history | 3542 | 2050 (58) | 0.97 (0.93-1.01) |
| **Child factors** | | | | |
| Survival status in HDSS | |  |  |  |
|  | Alive | 7086 | 4152 (59) | 1 (ref) |
|  | Dead before 5 years | 161 | 53 (33) | 0.56 (0.45-0.71) |
|  | Alive | 7086 | 4152 (59) | 1 (ref) |
|  | Dead day 0-6 | 38 | 14 (37) | 0.63 (0.41-0.97) |
|  | Dead day 7-27 | 30 | 8 (27) | 0.46 (0.25-0.83) |
|  | Dead day 28-364 | 53 | 15 (28) | 0.48 (0.31-0.76) |
|  | Dead 1-2 years | 35 | 14 (40) | 0.68 (0.46-1.02) |
|  | Dead 3-4 years | 5 | 2 (40) | 0.68 (0.23-2.00) |
| Sex | |  |  |  |
|  | Male | 2511 | 1649 (66) | 1 (ref) |
|  | Female | 2482 | 1617 (65) | 0.99 (0.95-1.03) |
|  | Missing | 2254 | 939 (42) |  |
| **Maternal factors** | | | | |
| Education | |  |  |  |
|  | None | 5059 | 2678 (53) | 1 (ref) |
|  | Primary school | 1399 | 905 (65) | 1.22 (1.16-1.29) |
|  | Secondary school | 386 | 296 (77) | 1.45 (1.36-1.54) |
|  | Higher education | 403 | 326 (81) | 1.53 (1.45-1.61) |
|  | Missing | 0 |  |  |
| Parity | |  |  |  |
|  | 1 | 478 | 325 (68) | 1 (ref) |
|  | 2 | 858 | 622 (72) | 1.07 (0.99-1.15) |
|  | 3 | 970 | 657 (68) | 1.00 (0.92-1.08) |
|  | 4 | 1154 | 693 (60) | 0.88 (0.81-0.96) |
|  | 5+ | 3699 | 1908 (52) | 0.76 (0.71-0.82) |
|  | Missing | 88 | 0 (0) |  |
| Wealth quintiles | |  |  |  |
|  | Poorest | 2284 | 1180 (52) | 1 (ref) |
|  | 2 | 1351 | 773 (57) | 1.11 (1.04-1.18) |
|  | 3 | 1499 | 862 (58) | 1.11 (1.04-1.19) |
|  | 4 | 1204 | 752 (62) | 1.21 (1.13-1.29) |
|  | Richest | 821 | 638 (78) | 1.50 (1.42-1.59) |
|  | Missing | 88 | 0 (0) |  |

### **Additional file 3.9C: Precision of survey estimates in capturing HDSS-recorded births limiting to births followed in HDSS since date-of-birth– IgangaMayuge**

|  |  | Number of children born to interviewed women under HDSS surveillance during the five years prior to EN-INDEPTH survey | Number of births (%) matching (+/-1 months) to a survey recorded birth | RR of capture in the survey (95%CI) |
| --- | --- | --- | --- | --- |
|  | All | 10133 | 5372 (53) |  |
| Recall period | |  |  |  |
|  | 0-1 years | 2459 | 1874 (76) | 1 (ref) |
|  | >2 years | 7674 | 3498 (46) | 0.60 (0.58-0.62) |
| **Survey tool** | | | | |
|  | Birth history | 5074 | 2652 (52) | 1 (ref) |
|  | Pregnancy history | 5059 | 2720 (54) | 1.03 (0.98-1.07) |
| **Child factors** | | | | |
| Survival status in HDSS | |  |  |  |
|  | Alive | 9794 | 5272 (54) | 1 (ref) |
|  | Dead before 5 years | 339 | 100 (29) | 0.55 (0.46-0.65) |
|  | Alive | 9794 | 5272 (54) | 1 (ref) |
|  | Dead day 0-6 | 125 | 35 (28) | 0.52 (0.38-0.71) |
|  | Dead day 7-27 | 25 | 11 (44) | 0.82 (0.51-1.32) |
|  | Dead day 28-364 | 64 | 28 (44) | 0.81 (0.61-1.09) |
|  | Dead 1-2 years | 98 | 21 (21) | 0.40 (0.27-0.59) |
|  | Dead 3-4 years | 29 | 7 (24) | 0.45 (0.24-0.85) |
| Sex | |  |  |  |
|  | Male | 5156 | 2753 (53) | 1 (ref) |
|  | Female | 4977 | 2619 (53) | 0.99 (0.95-1.02) |
|  | Missing | 0 |  |  |
| **Maternal factors** | | | | |
| Education | |  |  |  |
|  | None | 1141 | 476 (42) | 1 (ref) |
|  | Primary school | 5908 | 3061 (52) | 1.24 (1.14-1.35) |
|  | Secondary school | 2702 | 1592 (59) | 1.41 (1.30-1.54) |
|  | Higher education | 382 | 243 (64) | 1.52 (1.35-1.72) |
|  | Missing | 0 |  |  |
| Parity | |  |  |  |
|  | 1 | 610 | 410 (67) | 1 (ref) |
|  | 2 | 905 | 626 (69) | 1.03 (0.95-1.11) |
|  | 3 | 1100 | 776 (71) | 1.05 (0.98-1.13) |
|  | 4 | 1276 | 801 (63) | 0.93 (0.86-1.01) |
|  | 5+ | 5992 | 2759 (46) | 0.69 (0.64-0.73) |
|  | Missing | 250 | 0 (0) |  |
| Wealth quintiles | |  |  |  |
|  | Poorest | 2926 | 1566 (54) | 1 (ref) |
|  | 2 | 2423 | 1277 (53) | 0.98 (0.93-1.05) |
|  | 3 | 1921 | 1075 (56) | 1.05 (0.98-1.11) |
|  | 4 | 1474 | 804 (55) | 1.02 (0.95-1.09) |
|  | Richest | 1139 | 650 (57) | 1.07 (0.99-1.15) |
|  | Missing | 250 | 0 (0) |  |

### **Additional file 3.9D: Precision of survey estimates in capturing HDSS-recorded births limiting to births followed in HDSS since date-of-birth– Matlab**

|  |  | Number of children born to interviewed women under HDSS surveillance during the five years prior to EN-INDEPTH survey | Number of births (%) matching (+/-1 months) to a survey recorded birth | RR of capture in the survey (95%CI) |
| --- | --- | --- | --- | --- |
|  | All | 30235 | 27098 (90) |  |
| Recall period | |  |  |  |
|  | 0-1 years | 8197 | 7890 (96) | 1 (ref) |
|  | >2 years | 22038 | 19208 (87) | 0.91 (0.90-0.91) |
| **Survey tool** | | | | |
|  | Birth history | 15251 | 13694 (90) | 1 (ref) |
|  | Pregnancy history | 14952 | 13404 (90) | 1.00 (0.99-1.01) |
| **Child factors** | | | | |
| Survival status in HDSS | |  |  |  |
|  | Alive | 29581 | 26629 (90) | 1 (ref) |
|  | Dead before 5 years | 654 | 469 (72) | 0.80 (0.76-0.84) |
|  | Alive | 29581 | 26629 (90) | 1 (ref) |
|  | Dead day 0-6 | 327 | 226 (69) | 0.77 (0.71-0.83) |
|  | Dead day 7-27 | 76 | 55 (72) | 0.80 (0.70-0.93) |
|  | Dead day 28-364 | 107 | 85 (79) | 0.88 (0.80-0.97) |
|  | Dead 1-2 years | 132 | 96 (73) | 0.81 (0.73-0.90) |
|  | Dead 3-4 years | 47 | 39 (83) | 0.92 (0.81-1.05) |
| Sex | |  |  |  |
|  | Male | 15108 | 13622 (90) | 1 (ref) |
|  | Female | 15127 | 13476 (89) | 0.99 (0.98-1.00) |
|  | Missing | 0 |  |  |
| **Maternal factors** | | | | |
| Education | |  |  |  |
|  | None | 1320 | 1092 (83) | 1 (ref) |
|  | Primary school | 5863 | 5007 (85) | 1.03 (1.00-1.06) |
|  | Secondary school | 19418 | 17581 (91) | 1.09 (1.07-1.12) |
|  | Higher education | 3633 | 3418 (94) | 1.14 (1.11-1.17) |
|  | Missing | 1 | 0 (0) |  |
| Parity | |  |  |  |
|  | 1 | 6260 | 5935 (95) | 1 (ref) |
|  | 2 | 11970 | 10868 (91) | 0.96 (0.95-0.97) |
|  | 3 | 7842 | 6885 (88) | 0.93 (0.92-0.94) |
|  | 4 | 2943 | 2449 (83) | 0.88 (0.86-0.89) |
|  | 5+ | 1188 | 961 (81) | 0.85 (0.83-0.88) |
|  | Missing | 32 | 0 (0) |  |
| Wealth quintiles | |  |  |  |
|  | Poorest | 6379 | 5547 (87) | 1 (ref) |
|  | 2 | 6113 | 5440 (89) | 1.02 (1.01-1.04) |
|  | 3 | 5953 | 5352 (90) | 1.03 (1.02-1.05) |
|  | 4 | 5900 | 5374 (91) | 1.05 (1.03-1.06) |
|  | Richest | 5858 | 5385 (92) | 1.06 (1.04-1.07) |
|  | Missing | 32 | 0 (0) |  |

### **Additional file 3.9E: Precision of survey estimates in capturing HDSS-recorded births limiting to births followed in HDSS since date-of-birth– Kintampo**

|  |  | Number of children born to interviewed women under HDSS surveillance during the five years prior to EN-INDEPTH survey | Number of births (%) matching (+/-1 months) to a survey recorded birth | RR of capture in the survey (95%CI) |
| --- | --- | --- | --- | --- |
|  | All | 15041 | 13221 (88) |  |
| Recall period | |  |  |  |
|  | 0-1 years | 3399 | 3159 (93) | 1 (ref) |
|  | >2 years | 11642 | 10062 (86) | 0.93 (0.92-0.94) |
| **Survey tool** | | | | |
|  | Birth history | 7461 | 6592 (88) | 1 (ref) |
|  | Pregnancy history | 7580 | 6629 (87) | 0.99 (0.98-1.00) |
| **Child factors** | | | | |
| Survival status in HDSS | |  |  |  |
|  | Alive | 14655 | 13015 (89) | 1 (ref) |
|  | Dead before 5 years | 386 | 206 (53) | 0.60 (0.55-0.66) |
|  | Alive | 14655 | 13015 (89) | 1 (ref) |
|  | Dead day 0-6 | 144 | 66 (46) | 0.52 (0.43-0.62) |
|  | Dead day 7-27 | 31 | 23 (74) | 0.84 (0.68-1.03) |
|  | Dead day 28-364 | 110 | 58 (53) | 0.59 (0.50-0.71) |
|  | Dead 1-2 years | 100 | 58 (58) | 0.65 (0.55-0.77) |
|  | Dead 3-4 years | 11 | 6 (55) | 0.61 (0.36-1.05) |
| Sex | |  |  |  |
|  | Male | 7721 | 6821 (88) | 1 (ref) |
|  | Female | 7320 | 6400 (87) | 0.99 (0.98-1.00) |
|  | Missing | 0 |  |  |
| **Maternal factors** | | | | |
| Education | |  |  |  |
|  | None | 6121 | 5377 (88) | 1 (ref) |
|  | Primary school | 8296 | 7329 (88) | 1.01 (0.99-1.02) |
|  | Secondary school | 466 | 386 (83) | 0.94 (0.90-0.99) |
|  | Higher education | 158 | 129 (82) | 0.93 (0.86-1.01) |
|  | Missing | 0 |  |  |
| Parity | |  |  |  |
|  | 1 | 1913 | 1593 (83) | 1 (ref) |
|  | 2 | 2537 | 2226 (88) | 1.05 (1.03-1.08) |
|  | 3 | 2622 | 2348 (90) | 1.08 (1.05-1.10) |
|  | 4 | 2354 | 2096 (89) | 1.07 (1.04-1.10) |
|  | 5+ | 5613 | 4958 (88) | 1.06 (1.04-1.09) |
|  | Missing | 2 | 0 (0) |  |
| Wealth quintiles | |  |  |  |
|  | Poorest | 3154 | 2766 (88) | 1 (ref) |
|  | 2 | 3035 | 2677 (88) | 1.01 (0.99-1.03) |
|  | 3 | 2991 | 2658 (89) | 1.01 (0.99-1.03) |
|  | 4 | 2939 | 2589 (88) | 1.00 (0.98-1.02) |
|  | Richest | 2920 | 2531 (87) | 0.99 (0.97-1.01) |
|  | Missing | 2 | 0 (0) |  |

### **Additional file 3.10A: Precision of survey estimates in capturing HDSS-recorded events using wider age matching criteria- Bandim**

|  |  | Number of children born to interviewed women under HDSS surveillance during the five years prior to EN-INDEPTH survey | Number of births (%) matching (+/-3 months) to a survey recorded birth | RR of capture in the survey (95%CI) |
| --- | --- | --- | --- | --- |
|  | All | 16191 | 14124 (87) |  |
| Recall period | |  |  |  |
|  | 0-1 years | 4887 | 4655 (95) | 1 (ref) |
|  | >2 years | 11304 | 9469 (84) | 0.88 (0.87-0.89) |
| **Survey tool** | | | | |
|  | Birth history | 8284 | 7256 (88) | 1 (ref) |
|  | Pregnancy history | 7907 | 6868 (87) | 0.99 (0.98-1.00) |
| **Child factors** | | | | |
| Survival status in HDSS | |  |  |  |
|  | Alive | 15438 | 13745 (89) | 1 (ref) |
|  | Dead before 5 years | 753 | 379 (50) | 0.57 (0.52-0.61) |
|  | Alive | 15438 | 13745 (89) | 1 (ref) |
|  | Dead day 0-6 | 431 | 187 (43) | 0.49 (0.44-0.55) |
|  | Dead day 7-27 | 48 | 30 (63) | 0.70 (0.56-0.88) |
|  | Dead day 28-364 | 176 | 116 (66) | 0.74 (0.66-0.83) |
|  | Dead 1-2 years | 112 | 71 (63) | 0.71 (0.62-0.82) |
|  | Dead 3-4 years | 28 | 12 (43) | 0.48 (0.31-0.74) |
| Sex | |  |  |  |
|  | Male | 8281 | 7242 (87) | 1 (ref) |
|  | Female | 7893 | 6879 (87) | 1.00 (0.98-1.01) |
|  | Missing | 17 | 3 (18) |  |
| **Maternal factors** | | | | |
| Education | |  |  |  |
|  | None | 5588 | 4626 (83) | 1 (ref) |
|  | Primary school | 4767 | 4175 (88) | 1.06 (1.04-1.08) |
|  | Secondary school | 4758 | 4405 (93) | 1.12 (1.10-1.14) |
|  | Higher education | 967 | 917 (95) | 1.15 (1.12-1.17) |
|  | Missing | 111 | 1 (1) |  |
| Parity | |  |  |  |
|  | 1 | 2415 | 2283 (95) | 1 (ref) |
|  | 2 | 3255 | 2946 (91) | 0.96 (0.94-0.97) |
|  | 3 | 3171 | 2827 (89) | 0.94 (0.93-0.96) |
|  | 4 | 2596 | 2210 (85) | 0.90 (0.88-0.92) |
|  | 5+ | 4644 | 3858 (83) | 0.88 (0.86-0.89) |
|  | Missing | 110 | 0 (0) |  |
| Wealth quintiles | |  |  |  |
|  | Poorest | 2898 | 2643 (91) | 1 (ref) |
|  | 2 | 2961 | 2657 (90) | 0.98 (0.97-1.00) |
|  | 3 | 3002 | 2642 (88) | 0.96 (0.95-0.98) |
|  | 4 | 3138 | 2742 (87) | 0.96 (0.94-0.98) |
|  | Richest | 4082 | 3440 (84) | 0.92 (0.91-0.94) |
|  | Missing | 110 | 0 (0) |  |

### **Additional file 3.10B: Precision of survey estimates in capturing HDSS-recorded events using wider age matching criteria– Dabat**

|  |  | Number of children born to interviewed women under HDSS surveillance during the five years prior to EN-INDEPTH survey | Number of births (%) matching (+/-3 months) to a survey recorded birth | RR of capture in the survey (95%CI) |
| --- | --- | --- | --- | --- |
|  | All | 7999 | 5011 (63) |  |
| Recall period | |  |  |  |
|  | 0-1 years | 996 | 780 (78) | 1 (ref) |
|  | >2 years | 7003 | 4231 (60) | 0.77 (0.74-0.80) |
| **Survey tool** | | | | |
|  | Birth history | 3976 | 2553 (64) | 1 (ref) |
|  | Pregnancy history | 3928 | 2458 (63) | 0.97 (0.94-1.01) |
| **Child factors** | | | | |
| Survival status in HDSS | |  |  |  |
|  | Alive | 7833 | 4950 (63) | 1 (ref) |
|  | Dead before 5 years | 166 | 61 (37) | 0.58 (0.47-0.72) |
|  | Alive | 7833 | 4950 (63) | 1 (ref) |
|  | Dead day 0-6 | 38 | 15 (39) | 0.62 (0.41-0.94) |
|  | Dead day 7-27 | 30 | 9 (30) | 0.47 (0.27-0.83) |
|  | Dead day 28-364 | 54 | 18 (33) | 0.53 (0.36-0.78) |
|  | Dead 1-2 years | 39 | 17 (44) | 0.69 (0.48-0.99) |
|  | Dead 3-4 years | 5 | 2 (40) | 0.63 (0.22-1.85) |
| Sex | |  |  |  |
|  | Male | 2716 | 1893 (70) | 1 (ref) |
|  | Female | 2706 | 1899 (70) | 1.01 (0.97-1.04) |
|  | Missing | 2577 | 1219 (47) |  |
| **Maternal factors** | | | | |
| Education | |  |  |  |
|  | None | 5497 | 3165 (58) | 1 (ref) |
|  | Primary school | 1571 | 1081 (69) | 1.20 (1.14-1.25) |
|  | Secondary school | 454 | 361 (80) | 1.38 (1.31-1.46) |
|  | Higher education | 477 | 404 (85) | 1.47 (1.40-1.54) |
|  | Missing | 0 |  |  |
| Parity | |  |  |  |
|  | 1 | 534 | 386 (72) | 1 (ref) |
|  | 2 | 980 | 750 (77) | 1.06 (0.99-1.13) |
|  | 3 | 1125 | 818 (73) | 1.01 (0.94-1.08) |
|  | 4 | 1250 | 798 (64) | 0.88 (0.82-0.95) |
|  | 5+ | 4015 | 2259 (56) | 0.78 (0.73-0.83) |
|  | Missing | 95 | 0 (0) |  |
| Wealth quintiles | |  |  |  |
|  | Poorest | 2453 | 1386 (57) | 1 (ref) |
|  | 2 | 1508 | 918 (61) | 1.08 (1.02-1.14) |
|  | 3 | 1634 | 1028 (63) | 1.11 (1.05-1.18) |
|  | 4 | 1343 | 893 (66) | 1.18 (1.11-1.24) |
|  | Richest | 966 | 786 (81) | 1.44 (1.37-1.51) |
|  | Missing | 95 | 0 (0) |  |

### **Additional file 3.10C: Precision of survey estimates in capturing HDSS-recorded events using wider age matching criteria– IgangaMayuge**

|  |  | Number of children born to interviewed women under HDSS surveillance during the five years prior to EN-INDEPTH survey | Number of births (%) matching (+/-3 months) to a survey recorded birth | RR of capture in the survey (95%CI) |
| --- | --- | --- | --- | --- |
|  | All | 12401 | 7230 (58) |  |
| Recall period | |  |  |  |
|  | 0-1 years | 2508 | 2034 (81) | 1 (ref) |
|  | >2 years | 9893 | 5196 (53) | 0.65 (0.63-0.67) |
| **Survey tool** | | | | |
|  | Birth history | 6259 | 3593 (57) | 1 (ref) |
|  | Pregnancy history | 6142 | 3637 (59) | 1.03 (1.00-1.07) |
| **Child factors** | | | | |
| Survival status in HDSS | |  |  |  |
|  | Alive | 12049 | 7111 (59) | 1 (ref) |
|  | Dead before 5 years | 352 | 119 (34) | 0.57 (0.49-0.67) |
|  | Alive | 12049 | 7111 (59) | 1 (ref) |
|  | Dead day 0-6 | 125 | 43 (34) | 0.58 (0.44-0.77) |
|  | Dead day 7-27 | 25 | 11 (44) | 0.75 (0.46-1.20) |
|  | Dead day 28-364 | 66 | 31 (47) | 0.80 (0.61-1.04) |
|  | Dead 1-2 years | 104 | 27 (26) | 0.44 (0.32-0.61) |
|  | Dead 3-4 years | 34 | 9 (26) | 0.45 (0.26-0.79) |
| Sex | |  |  |  |
|  | Male | 6276 | 3676 (59) | 1 (ref) |
|  | Female | 6125 | 3554 (58) | 0.99 (0.96-1.02) |
|  | Missing | 0 |  |  |
| **Maternal factors** | | | | |
| Education | |  |  |  |
|  | None | 1312 | 623 (47) | 1 (ref) |
|  | Primary school | 7002 | 4060 (58) | 1.22 (1.14-1.31) |
|  | Secondary school | 3554 | 2200 (62) | 1.30 (1.22-1.40) |
|  | Higher education | 533 | 347 (65) | 1.37 (1.25-1.51) |
|  | Missing | 0 |  |  |
| Parity | |  |  |  |
|  | 1 | 712 | 489 (69) | 1 (ref) |
|  | 2 | 1222 | 850 (70) | 1.01 (0.95-1.08) |
|  | 3 | 1509 | 1067 (71) | 1.03 (0.96-1.10) |
|  | 4 | 1668 | 1104 (66) | 0.96 (0.90-1.03) |
|  | 5+ | 6966 | 3720 (53) | 0.78 (0.73-0.83) |
|  | Missing | 324 | 0 (0) |  |
| Wealth quintiles | |  |  |  |
|  | Poorest | 3438 | 2036 (59) | 1 (ref) |
|  | 2 | 2819 | 1677 (59) | 1.00 (0.96-1.05) |
|  | 3 | 2289 | 1391 (61) | 1.03 (0.98-1.08) |
|  | 4 | 1941 | 1136 (59) | 0.99 (0.94-1.04) |
|  | Richest | 1590 | 990 (62) | 1.05 (0.99-1.11) |
|  | Missing | 324 | 0 (0) |  |

### **Additional file 3.10D: Precision of survey estimates in capturing HDSS-recorded events using wider age matching criteria- Matlab**

|  |  | Number of children born to interviewed women under HDSS surveillance during the five years prior to EN-INDEPTH survey | Number of births (%) matching (+/-3 months) to a survey recorded birth | RR of capture in the survey (95%CI) |
| --- | --- | --- | --- | --- |
|  | All | 31992 | 29240 (91) |  |
| Recall period | |  |  |  |
|  | 0-1 years | 8240 | 8029 (97) | 1 (ref) |
|  | >2 years | 23752 | 21211 (89) | 0.92 (0.91-0.92) |
| **Survey tool** | | | | |
|  | Birth history | 16122 | 14796 (92) | 1 (ref) |
|  | Pregnancy history | 15799 | 14444 (91) | 1.00 (0.99-1.00) |
| **Child factors** | | | | |
| Survival status in HDSS | |  |  |  |
|  | Alive | 31324 | 28728 (92) | 1 (ref) |
|  | Dead before 5 years | 668 | 512 (77) | 0.84 (0.80-0.87) |
|  | Alive | 31324 | 28728 (92) | 1 (ref) |
|  | Dead day 0-6 | 327 | 244 (75) | 0.81 (0.76-0.87) |
|  | Dead day 7-27 | 76 | 60 (79) | 0.86 (0.76-0.97) |
|  | Dead day 28-364 | 108 | 93 (86) | 0.94 (0.87-1.01) |
|  | Dead 1-2 years | 141 | 109 (77) | 0.84 (0.77-0.92) |
|  | Dead 3-4 years | 51 | 39 (76) | 0.83 (0.72-0.97) |
| Sex | |  |  |  |
|  | Male | 15935 | 14648 (92) | 1 (ref) |
|  | Female | 16057 | 14592 (91) | 0.99 (0.98-1.00) |
|  | Missing | 0 |  |  |
| **Maternal factors** | | | | |
| Education | |  |  |  |
|  | None | 1385 | 1183 (85) | 1 (ref) |
|  | Primary school | 6248 | 5496 (88) | 1.03 (1.00-1.06) |
|  | Secondary school | 20510 | 18920 (92) | 1.08 (1.05-1.11) |
|  | Higher education | 3848 | 3641 (95) | 1.11 (1.08-1.14) |
|  | Missing | 1 | 0 (0) |  |
| Parity | |  |  |  |
|  | 1 | 6330 | 6079 (96) | 1 (ref) |
|  | 2 | 12909 | 11911 (92) | 0.96 (0.95-0.97) |
|  | 3 | 8353 | 7529 (90) | 0.94 (0.93-0.95) |
|  | 4 | 3092 | 2671 (86) | 0.90 (0.89-0.91) |
|  | 5+ | 1237 | 1050 (85) | 0.88 (0.86-0.91) |
|  | Missing | 71 | 0 (0) |  |
| Wealth quintiles | |  |  |  |
|  | Poorest | 6763 | 6070 (90) | 1 (ref) |
|  | 2 | 6405 | 5831 (91) | 1.01 (1.00-1.03) |
|  | 3 | 6293 | 5790 (92) | 1.03 (1.01-1.04) |
|  | 4 | 6212 | 5728 (92) | 1.03 (1.02-1.04) |
|  | Richest | 6248 | 5821 (93) | 1.04 (1.03-1.05) |
|  | Missing | 71 | 0 (0) |  |

### **Additional file 3.10E: Precision of survey estimates in capturing HDSS-recorded events using wider age matching criteria– Kintampo**

|  |  | Number of children born to interviewed women under HDSS surveillance during the five years prior to EN-INDEPTH survey | Number of births (%) matching (+/-3 months) to a survey recorded birth | RR of capture in the survey (95%CI) |
| --- | --- | --- | --- | --- |
|  | All | 15185 | 13643 (90) |  |
| Recall period | |  |  |  |
|  | 0-1 years | 3399 | 3204 (94) | 1 (ref) |
|  | >2 years | 11786 | 10439 (89) | 0.94 (0.93-0.95) |
| **Survey tool** | | | | |
|  | Birth history | 7535 | 6800 (90) | 1 (ref) |
|  | Pregnancy history | 7650 | 6843 (89) | 0.99 (0.98-1.00) |
| **Child factors** | | | | |
| Survival status in HDSS | |  |  |  |
|  | Alive | 14799 | 13427 (91) | 1 (ref) |
|  | Dead before 5 years | 386 | 216 (56) | 0.62 (0.56-0.68) |
|  | Alive | 14799 | 13427 (91) | 1 (ref) |
|  | Dead day 0-6 | 144 | 72 (50) | 0.55 (0.47-0.65) |
|  | Dead day 7-27 | 31 | 23 (74) | 0.82 (0.67-1.00) |
|  | Dead day 28-364 | 110 | 59 (54) | 0.59 (0.49-0.71) |
|  | Dead 1-2 years | 100 | 62 (62) | 0.68 (0.59-0.80) |
|  | Dead 3-4 years | 11 | 6 (55) | 0.60 (0.35-1.03) |
| Sex | |  |  |  |
|  | Male | 7790 | 7030 (90) | 1 (ref) |
|  | Female | 7395 | 6613 (89) | 0.99 (0.98-1.00) |
|  | Missing | 0 |  |  |
| **Maternal factors** | | | | |
| Education | |  |  |  |
|  | None | 6183 | 5566 (90) | 1 (ref) |
|  | Primary school | 8363 | 7547 (90) | 1.00 (0.99-1.01) |
|  | Secondary school | 478 | 399 (83) | 0.93 (0.89-0.97) |
|  | Higher education | 161 | 131 (81) | 0.90 (0.83-0.98) |
|  | Missing | 0 |  |  |
| Parity | |  |  |  |
|  | 1 | 1930 | 1626 (84) | 1 (ref) |
|  | 2 | 2564 | 2297 (90) | 1.06 (1.04-1.09) |
|  | 3 | 2647 | 2429 (92) | 1.09 (1.06-1.12) |
|  | 4 | 2384 | 2165 (91) | 1.08 (1.05-1.10) |
|  | 5+ | 5658 | 5126 (91) | 1.08 (1.05-1.10) |
|  | Missing | 2 | 0 (0) |  |
| Wealth quintiles | |  |  |  |
|  | Poorest | 3180 | 2856 (90) | 1 (ref) |
|  | 2 | 3064 | 2766 (90) | 1.01 (0.99-1.02) |
|  | 3 | 3020 | 2739 (91) | 1.01 (0.99-1.03) |
|  | 4 | 2969 | 2672 (90) | 1.00 (0.98-1.02) |
|  | Richest | 2950 | 2610 (88) | 0.99 (0.97-1.00) |
|  | Missing | 2 | 0 (0) |  |

### **Additional file 3.11A: Precision of survey estimates in capturing HDSS-recorded events using wider age matching criteria- Bandim**

|  |  | Number of children born to interviewed women under HDSS surveillance during the five years prior to EN-INDEPTH survey | Number of births (%) matching (+/-9 months) to a survey recorded birth | RR of capture in the survey (95%CI) |
| --- | --- | --- | --- | --- |
|  | All | 16191 | 14524 (90) |  |
| Recall period | |  |  |  |
|  | 0-1 years | 4887 | 4695 (96) | 1 (ref) |
|  | >2 years | 11304 | 9829 (87) | 0.91 (0.90-0.91) |
| **Survey tool** | | | | |
|  | Birth history | 8284 | 7463 (90) | 1 (ref) |
|  | Pregnancy history | 7907 | 7061 (89) | 0.99 (0.98-1.00) |
| **Child factors** | | | | |
| Survival status in HDSS | |  |  |  |
|  | Alive | 15438 | 14122 (91) | 1 (ref) |
|  | Dead before 5 years | 753 | 402 (53) | 0.58 (0.54-0.63) |
|  | Alive | 15438 | 14122 (91) | 1 (ref) |
|  | Dead day 0-6 | 431 | 196 (45) | 0.50 (0.45-0.55) |
|  | Dead day 7-27 | 48 | 31 (65) | 0.71 (0.57-0.87) |
|  | Dead day 28-364 | 176 | 125 (71) | 0.78 (0.71-0.85) |
|  | Dead 1-2 years | 112 | 74 (66) | 0.72 (0.63-0.83) |
|  | Dead 3-4 years | 28 | 15 (54) | 0.59 (0.41-0.83) |
| Sex | |  |  |  |
|  | Male | 8281 | 7432 (90) | 1 (ref) |
|  | Female | 7893 | 7089 (90) | 1.00 (0.99-1.01) |
|  | Missing | 17 | 3 (18) |  |
| **Maternal factors** | | | | |
| Education | |  |  |  |
|  | None | 5588 | 4876 (87) | 1 (ref) |
|  | Primary school | 4767 | 4275 (90) | 1.03 (1.01-1.04) |
|  | Secondary school | 4758 | 4450 (94) | 1.07 (1.06-1.09) |
|  | Higher education | 967 | 922 (95) | 1.09 (1.07-1.11) |
|  | Missing | 111 | 1 (1) |  |
| Parity | |  |  |  |
|  | 1 | 2415 | 2300 (95) | 1 (ref) |
|  | 2 | 3255 | 3007 (92) | 0.97 (0.96-0.98) |
|  | 3 | 3171 | 2895 (91) | 0.96 (0.94-0.97) |
|  | 4 | 2596 | 2295 (88) | 0.93 (0.91-0.95) |
|  | 5+ | 4644 | 4027 (87) | 0.91 (0.90-0.92) |
|  | Missing | 110 | 0 (0) |  |
| Wealth quintiles | |  |  |  |
|  | Poorest | 2898 | 2682 (93) | 1 (ref) |
|  | 2 | 2961 | 2721 (92) | 0.99 (0.98-1.01) |
|  | 3 | 3002 | 2721 (91) | 0.98 (0.96-1.00) |
|  | 4 | 3138 | 2834 (90) | 0.98 (0.96-0.99) |
|  | Richest | 4082 | 3566 (87) | 0.94 (0.93-0.96) |
|  | Missing | 110 | 0 (0) |  |

### **Additional file 3.11B: Precision of survey estimates in capturing HDSS-recorded events using wider age matching criteria- Dabat**

|  |  | Number of children born to interviewed women under HDSS surveillance during the five years prior to EN-INDEPTH survey | Number of births (%) matching (+/-9 months) to a survey recorded birth | RR of capture in the survey (95%CI) |
| --- | --- | --- | --- | --- |
|  | All | 7999 | 5463 (68) |  |
| Recall period | |  |  |  |
|  | 0-1 years | 996 | 814 (82) | 1 (ref) |
|  | >2 years | 7003 | 4649 (66) | 0.81 (0.78-0.84) |
| **Survey tool** | | | | |
|  | Birth history | 3976 | 2777 (70) | 1 (ref) |
|  | Pregnancy history | 3928 | 2686 (68) | 0.98 (0.95-1.01) |
| **Child factors** | | | | |
| Survival status in HDSS | |  |  |  |
|  | Alive | 7833 | 5396 (69) | 1 (ref) |
|  | Dead before 5 years | 166 | 67 (40) | 0.59 (0.48-0.71) |
|  | Alive | 7833 | 5396 (69) | 1 (ref) |
|  | Dead day 0-6 | 38 | 17 (45) | 0.65 (0.45-0.94) |
|  | Dead day 7-27 | 30 | 10 (33) | 0.48 (0.29-0.81) |
|  | Dead day 28-364 | 54 | 18 (33) | 0.48 (0.33-0.72) |
|  | Dead 1-2 years | 39 | 19 (49) | 0.71 (0.51-0.98) |
|  | Dead 3-4 years | 5 | 3 (60) | 0.87 (0.43-1.78) |
| Sex | |  |  |  |
|  | Male | 2716 | 2012 (74) | 1 (ref) |
|  | Female | 2706 | 2021 (75) | 1.01 (0.98-1.04) |
|  | Missing | 2577 | 1430 (55) |  |
| **Maternal factors** | | | | |
| Education | |  |  |  |
|  | None | 5497 | 3517 (64) | 1 (ref) |
|  | Primary school | 1571 | 1158 (74) | 1.15 (1.11-1.20) |
|  | Secondary school | 454 | 372 (82) | 1.28 (1.22-1.35) |
|  | Higher education | 477 | 416 (87) | 1.36 (1.31-1.42) |
|  | Missing | 0 |  |  |
| Parity | |  |  |  |
|  | 1 | 534 | 406 (76) | 1 (ref) |
|  | 2 | 980 | 787 (80) | 1.06 (0.99-1.12) |
|  | 3 | 1125 | 854 (76) | 1.00 (0.94-1.06) |
|  | 4 | 1250 | 860 (69) | 0.90 (0.85-0.96) |
|  | 5+ | 4015 | 2556 (64) | 0.84 (0.79-0.89) |
|  | Missing | 95 | 0 (0) |  |
| Wealth quintiles | |  |  |  |
|  | Poorest | 2453 | 1571 (64) | 1 (ref) |
|  | 2 | 1508 | 1004 (67) | 1.04 (0.99-1.09) |
|  | 3 | 1634 | 1112 (68) | 1.06 (1.01-1.11) |
|  | 4 | 1343 | 955 (71) | 1.11 (1.06-1.16) |
|  | Richest | 966 | 821 (85) | 1.33 (1.27-1.38) |
|  | Missing | 95 | 0 (0) |  |

### **Additional file 3.11C: Precision of survey estimates in capturing HDSS-recorded events using wider age matching criteria– IgangaMayuge**

|  |  | Number of children born to interviewed women under HDSS surveillance during the five years prior to EN-INDEPTH survey | Number of births (%) matching (+/-9 months) to a survey recorded birth | RR of capture in the survey (95%CI) |
| --- | --- | --- | --- | --- |
|  | All | 12401 | 8973 (72) |  |
| Recall period | |  |  |  |
|  | 0-1 years | 2508 | 2172 (87) | 1 (ref) |
|  | >2 years | 9893 | 6801 (69) | 0.79 (0.78-0.81) |
| **Survey tool** | | | | |
|  | Birth history | 6259 | 4508 (72) | 1 (ref) |
|  | Pregnancy history | 6142 | 4465 (73) | 1.01 (0.99-1.03) |
| **Child factors** | | | | |
| Survival status in HDSS | |  |  |  |
|  | Alive | 12049 | 8808 (73) | 1 (ref) |
|  | Dead before 5 years | 352 | 165 (47) | 0.64 (0.57-0.72) |
|  | Alive | 12049 | 8808 (73) | 1 (ref) |
|  | Dead day 0-6 | 125 | 53 (42) | 0.58 (0.46-0.73) |
|  | Dead day 7-27 | 25 | 12 (48) | 0.66 (0.42-1.02) |
|  | Dead day 28-364 | 66 | 38 (58) | 0.79 (0.64-0.97) |
|  | Dead 1-2 years | 104 | 45 (43) | 0.59 (0.47-0.74) |
|  | Dead 3-4 years | 34 | 19 (56) | 0.76 (0.57-1.03) |
| Sex | |  |  |  |
|  | Male | 6276 | 4573 (73) | 1 (ref) |
|  | Female | 6125 | 4400 (72) | 0.99 (0.96-1.01) |
|  | Missing | 0 |  |  |
| **Maternal factors** | | | | |
| Education | |  |  |  |
|  | None | 1312 | 856 (65) | 1 (ref) |
|  | Primary school | 7002 | 5057 (72) | 1.11 (1.06-1.16) |
|  | Secondary school | 3554 | 2652 (75) | 1.14 (1.09-1.20) |
|  | Higher education | 533 | 408 (77) | 1.17 (1.10-1.26) |
|  | Missing | 0 |  |  |
| Parity | |  |  |  |
|  | 1 | 712 | 543 (76) | 1 (ref) |
|  | 2 | 1222 | 981 (80) | 1.05 (1.00-1.11) |
|  | 3 | 1509 | 1227 (81) | 1.07 (1.01-1.12) |
|  | 4 | 1668 | 1307 (78) | 1.03 (0.97-1.08) |
|  | 5+ | 6966 | 4915 (71) | 0.93 (0.88-0.97) |
|  | Missing | 324 | 0 (0) |  |
| Wealth quintiles | |  |  |  |
|  | Poorest | 3438 | 2550 (74) | 1 (ref) |
|  | 2 | 2819 | 2061 (73) | 0.99 (0.95-1.02) |
|  | 3 | 2289 | 1733 (76) | 1.02 (0.99-1.05) |
|  | 4 | 1941 | 1420 (73) | 0.99 (0.95-1.02) |
|  | Richest | 1590 | 1209 (76) | 1.03 (0.99-1.06) |
|  | Missing | 324 | 0 (0) |  |

### **Additional file 3.11D: Precision of survey estimates in capturing HDSS-recorded events using wider age matching criteria- Matlab**

|  |  | Number of children born to interviewed women under HDSS surveillance during the five years prior to EN-INDEPTH survey | Number of births (%) matching (+/-9 months) to a survey recorded birth | RR of capture in the survey (95%CI) |
| --- | --- | --- | --- | --- |
|  | All | 31992 | 29702 (93) |  |
| Recall period | |  |  |  |
|  | 0-1 years | 8240 | 8076 (98) | 1 (ref) |
|  | >2 years | 23752 | 21626 (91) | 0.93 (0.92-0.93) |
| **Survey tool** | | | | |
|  | Birth history | 16122 | 15026 (93) | 1 (ref) |
|  | Pregnancy history | 15799 | 14676 (93) | 1.00 (0.99-1.00) |
| **Child factors** | | | | |
| Survival status in HDSS | |  |  |  |
|  | Alive | 31324 | 29161 (93) | 1 (ref) |
|  | Dead before 5 years | 668 | 541 (81) | 0.87 (0.84-0.90) |
|  | Alive | 31324 | 29161 (93) | 1 (ref) |
|  | Dead day 0-6 | 327 | 266 (81) | 0.87 (0.83-0.92) |
|  | Dead day 7-27 | 76 | 61 (80) | 0.86 (0.77-0.97) |
|  | Dead day 28-364 | 108 | 95 (88) | 0.94 (0.88-1.01) |
|  | Dead 1-2 years | 141 | 112 (79) | 0.85 (0.78-0.93) |
|  | Dead 3-4 years | 51 | 40 (78) | 0.84 (0.73-0.97) |
| Sex | |  |  |  |
|  | Male | 15935 | 14858 (93) | 1 (ref) |
|  | Female | 16057 | 14844 (92) | 0.99 (0.99-1.00) |
|  | Missing | 0 |  |  |
| **Maternal factors** | | | | |
| Education | |  |  |  |
|  | None | 1385 | 1223 (88) | 1 (ref) |
|  | Primary school | 6248 | 5642 (90) | 1.02 (1.00-1.05) |
|  | Secondary school | 20510 | 19176 (93) | 1.06 (1.04-1.08) |
|  | Higher education | 3848 | 3661 (95) | 1.08 (1.05-1.10) |
|  | Missing | 1 | 0 (0) |  |
| Parity | |  |  |  |
|  | 1 | 6330 | 6119 (97) | 1 (ref) |
|  | 2 | 12909 | 12055 (93) | 0.97 (0.96-0.97) |
|  | 3 | 8353 | 7674 (92) | 0.95 (0.94-0.96) |
|  | 4 | 3092 | 2762 (89) | 0.92 (0.91-0.94) |
|  | 5+ | 1237 | 1092 (88) | 0.91 (0.89-0.93) |
|  | Missing | 71 | 0 (0) |  |
| Wealth quintiles | |  |  |  |
|  | Poorest | 6763 | 6196 (92) | 1 (ref) |
|  | 2 | 6405 | 5939 (93) | 1.01 (1.00-1.02) |
|  | 3 | 6293 | 5898 (94) | 1.02 (1.01-1.03) |
|  | 4 | 6212 | 5801 (93) | 1.02 (1.01-1.03) |
|  | Richest | 6248 | 5868 (94) | 1.03 (1.01-1.04) |
|  | Missing | 71 | 0 (0) |  |

### **Additional file 3.11E: Precision of survey estimates in capturing HDSS-recorded events using wider age matching criteria- Kintampo**

|  |  | Number of children born to interviewed women under HDSS surveillance during the five years prior to EN-INDEPTH survey | Number of births (%) matching (+/-9 months) to a survey recorded birth | RR of capture in the survey (95%CI) |  |
| --- | --- | --- | --- | --- | --- |
|  | All | 15185 | 14070 (93) |  |  |
| Recall period | |  |  |  |  |
|  | 0-1 years | 3399 | 3255 (96) | 1 (ref) |  |
|  | >2 years | 11786 | 10815 (92) | 0.96 (0.95-0.97) |  |
| **Survey tool** | | | | | |
|  | Birth history | 7535 | 7003 (93) | 1 (ref) |  |
|  | Pregnancy history | 7650 | 7067 (92) | 0.99 (0.98-1.00) |  |
| **Child factors** | | | | | |
| Survival status in HDSS | |  |  |  |  |
|  | Alive | 14799 | 13821 (93) | 1 (ref) |  |
|  | Dead before 5 years | 386 | 249 (65) | 0.69 (0.64-0.74) |  |
|  | Alive | 14799 | 13821 (93) | 1 (ref) |  |
|  | Dead day 0-6 | 144 | 84 (58) | 0.62 (0.54-0.72) |  |
|  | Dead day 7-27 | 31 | 25 (81) | 0.86 (0.73-1.02) |  |
|  | Dead day 28-364 | 110 | 70 (64) | 0.68 (0.59-0.79) |  |
|  | Dead 1-2 years | 100 | 70 (70) | 0.75 (0.66-0.85) |  |
|  | Dead 3-4 years | 11 | 6 (55) | 0.58 (0.34-1.00) |  |
| Sex | |  |  |  |  |
|  | Male | 7790 | 7228 (93) | 1 (ref) |  |
|  | Female | 7395 | 6842 (93) | 1.00 (0.99-1.01) |  |
|  | Missing | 0 |  |  |  |
| **Maternal factors** | | | | | |
| Education | |  |  |  |  |
|  | None | 6183 | 5772 (93) | 1 (ref) |  |
|  | Primary school | 8363 | 7749 (93) | 0.99 (0.98-1.00) |  |
|  | Secondary school | 478 | 416 (87) | 0.93 (0.90-0.97) |  |
|  | Higher education | 161 | 133 (83) | 0.88 (0.82-0.96) |  |
|  | Missing | 0 |  |  |  |
| Parity | |  |  |  |  |
|  | 1 | 1930 | 1664 (86) | 1 (ref) |  |
|  | 2 | 2564 | 2356 (92) | 1.07 (1.04-1.09) |  |
|  | 3 | 2647 | 2490 (94) | 1.09 (1.07-1.11) |  |
|  | 4 | 2384 | 2254 (95) | 1.10 (1.07-1.12) |  |
|  | 5+ | 5658 | 5306 (94) | 1.09 (1.07-1.11) |  |
|  | Missing | 2 | 0 (0) |  |  |
| Wealth quintiles | |  |  |  |  |
|  | Poorest | 3180 | 2966 (93) | 1 (ref) |  |
|  | 2 | 3064 | 2852 (93) | 1.00 (0.98-1.01) |  |
|  | 3 | 3020 | 2813 (93) | 1.00 (0.98-1.01) |  |
|  | 4 | 2969 | 2756 (93) | 1.00 (0.98-1.01) |  |
|  | Richest | 2950 | 2683 (91) | 0.98 (0.96-0.99) |  |
|  | Missing | 2 | 0 (0) |  |  |
