# Supplementary material for: Neonatal and child mortality data in retrospective population-based surveys compared with prospective demographic surveillance: EN-INDEPTH study
Source: Popul Health Metr. 2021 Feb 8;19(Suppl 1):7. doi: 10.1186/s12963-020-00232-1 (PMC7869220; doi:10.1186/s12963-020-00232-1)
Supplement: Supplementary file 4 — Additional file 4. Ethical approval of local Institutional Review Boards. [file 12963_2020_232_MOESM4_ESM.docx]

## **Additional file 4: Ethical approval of local Institutional Review Boards**

| **HDSS site** | **Institutional Review Boards** | **Date** | **Number/Ref** |
| --- | --- | --- | --- |
| Bandim | Comité Nacional de Ética na Saúde | 12 June 2017 | 072/CNES/INASA/2017 |
| Dabat | Institutional Review Board, University of Gondar | 19 April 2017 | VP/RCS/05/1074/2016 |
| IgangaMayuge | Mildmay Uganda Research Ethics Committee  Uganda National Council of Science and Technology | 26 June 2017  11 October 2017 | REC REF 0305-2017  SS 4244 |
| Kintampo | Kintampo Health Research Centre, Ghana Health service  Ghana Health Services Ethics Review Committee  Kintampo Health Research Centre Institutional Ethics Committee | 14 June 2017  26 July 2017  9 August 2017 | SRC/130617  GHS-ERC:19/06/14  KHRCIEC/2017-14 |
| Matlab | icddr,b Ethical Review Committee | 19 July 2017 | PR-17049 |
| London School of Hygiene & Tropical Medicine | London School of Hygiene & Tropical Medicine | 24 May 2017 | 12218 |
